# Lessons from the COVID-19 pandemic to strengthen NCD care and policy in humanitarian settings: a mixed methods study exploring humanitarian actors’ experiences

**DOI:** 10.1186/s12913-024-11458-2

**Published:** 2024-09-17

**Authors:** Éimhín Ansbro, Olivia Heller, Lavanya Vijayasingham, Caroline Favas, Jacqueline Rintjema, Alyssa Chase-Vilchez, Claire Stein, Rita Issa, Leah Sanga, Adrianna Murphy, Pablo Perel

**Affiliations:** 1 Department of Epidemiology of Noncommunicable Diseases, Epidemiology & Population Health, London School of Hygiene & Tropical Medicine, London, UK; 2 Centre for Global Chronic Conditions, London, School of Hygiene & Tropical Medicine , London, UK; 3 Service de Médecine Tropicale Et Humanitaire, Hôpitaux Universitaires de Genève, Geneva, Switzerland; 4 Faculty of Law, University of Toronto, Toronto, Canada; 5 Global Alliance for Chronic Diseases, Wellcome, London, UK; 6 Help Age International, Yangon, Myanmar; 7 Faculty of Economics and Business, University of Groningen, Groningen, The Netherlands; 8 School of Global Development, University of East Anglia, Norwich, UK; 9 T.H. Chan School of Public Health, FXB Center for Health and Human Rights, Harvard University, Boston, USA; 10 Department of Health Services Research and Policy, Public Health & Policy, London School of Hygiene & Tropical Medicine, London, UK

**Keywords:** Humanitarian, COVID-19, Pandemic, Noncommunicable, Implementation, Hypertension, Diabetes, Crisis, Disaster, Service delivery

## Abstract

**Background:**

The COVID-19 pandemic and response severely impacted people living with non-communicable diseases (PLWNCDs) globally. It exacerbated pre-existing health inequalities, severely disrupted access to care, and worsened clinical outcomes for PLWNCDs, who were at higher risk of morbidity and mortality from the virus. The pandemic’s effects were likely magnified in humanitarian settings, where there were pre-existing gaps in continuity of care for non-communicable diseases (NCDs). We sought to explore factors affecting implementation of NCD care in crisis settings during the COVID-19 pandemic and the adaptations made to support implementation.

**Methods:**

Guided by the Consolidated Framework for Implementation Research, we undertook an online survey of 98 humanitarian actors from multiple regions and organization types (March-July 2021), followed by in-depth interviews with 13 purposively selected survey respondents (October-December 2021)*.* Survey data were analysed using descriptive statistics, while interview data were analysed thematically, using both deductive and inductive approaches.

**Results:**

Initially, humanitarian actors faced challenges influenced by external actors’ priorities, such as de-prioritisation of NCD care by governments, travel restrictions and supply chain interruptions. With each infection wave and lockdown, humanitarian actors were better able to adapt and maintain NCD services. The availability of COVID-19 vaccines was a positive turning point, especially for the risk management of people with NCDs and protection of health workers. Key findings include that, despite pre-existing challenges, humanitarian actors largely continued NCD services during the crisis. Enabling factors that supported continuity of NCD services included the ability to quickly pivot to remote means of communication with PLWNCDs, flexibility in medicine dispensing, and successful advocacy to prioritize NCD management within health systems. Key lessons learned included the importance of partnerships and cooperation with other health actors, and the mobilisation or repurposing of community health workers/volunteer networks.

**Conclusions:**

The COVID-19 experience should prompt national and global health stakeholders to strengthen inclusion of NCDs in emergency preparedness, response, and resilience planning. Key lessons were learned around remote care provision, including adapting to NCD severity, integrating community health workers, providing context-adapted patient information, combating misinformation, and strengthening cross-sectoral partnerships.

**Supplementary Information:**

The online version contains supplementary material available at 10.1186/s12913-024-11458-2.

## Background

The SARS-2 Coronavirus (COVID-19) pandemic caused unprecedented challenges worldwide, testing healthcare systems across continents, affecting populations’ health and wellbeing, and highlighting global and national inequities [1, 2]. COVID-19 was more likely to cause severe infection and death in people who were older (75 years and above), immunocompromised or living with non-communicable disease (NCD) [1, 3]. As early as May 2020, NCDs and COVID-19 were cast as twin epidemics and later as a “syndemic.” They acted synergistically on morbidity and mortality, and shared a common set of underlying risk factors, including socio-economic deprivation, obesity, older age, and ethnicity [4]. As COVID-19 deaths reached one million worldwide, and the key roles of social inequity and failed political leadership were recognised, there was growing acknowledgement that tackling NCDs would be a “prerequisite for successful containment” of COVID-19 [5]. This required a broader syndemic approach, encompassing housing, education, employment, health, and the environmental sectors.

For decades before the pandemic, NCDs, notably cardiovascular diseases, cancers, diabetes, and chronic respiratory diseases, were the leading causes of mortality globally. They are responsible for 41 million deaths each year, equating to 75% of total global deaths [6]. People living in low and middle-income countries (LMICs), where the majority (70%) of global NCD deaths occur, are disproportionally affected by premature NCD mortality (i.e., deaths occurring before the age of 70) [6]. For best outcomes, people living with NCDs (PLWNCDs) require functioning health systems to deliver a continuum of care. This includes early detection through screening and diagnosis; accessible and continuous care and medications; and supported self-care and education, as well as context-adapted healthy eating and exercise opportunities [7, 8].

In parallel, more people than ever are affected by humanitarian crises, which have become more complex and prolonged [7–9]. Conflict, violence, and socio-economic inequity drive most of these crises, and many are now compounded by climate change. In 2021, COVID-19 overlaid other pre-existing and emerging crisis risks, as humanitarian needs remained at historically high levels. An estimated 306 million people were in need in 2021, 90.4 million more than in 2019, before the COVID-19 pandemic hit [10, 11].

Humanitarian emergencies disrupt care for NCDs, through destruction of health infrastructure and supply chains, and by reducing access to care. The continuum from diagnosis and screening services, to medical consultation, provision of regular medicines and equipment, and referral pathways may all be affected. Limited evidence also shows that the rates of acute exacerbations, including heart attacks, strokes, asthma attacks, and amputations are increased by stress, and are higher both during emergencies and in their immediate aftermath [7, 8]. Recent World Health Assembly resolutions and the World Health Organization (WHO) NCD Global Action Plan 2013–2030 underlined the importance of ensuring that refugees and internally displaced people can access care for NCDs [12]. However, until recently, NCDs have not been afforded the same priority as other important health concerns during acute crises, and have often been insufficiently integrated into emergency preparedness and response [13].

Refugees and other displaced people and those with limited health care access—as well as PLWNCDs—were considered “high burden” populations affected by the pandemic and its response [14]. Many national response policies to manage COVID-19 infections directly caused disruptions of NCD services along the continuum of care [15, 16]. A WHO survey conducted from May to July 2020 indicated that about 75% of global NCD services were disrupted in the early days of the pandemic, with low (65%) and lower- middle income (49%) countries most affected [17]. In the initial months of the pandemic in 2020, NCD care was commonly disrupted because of the urgent diversion of health care resources towards the COVID-19 response, government-imposed travel restrictions, advice to high-risk people to isolate, and people’s understandable fear of attending health facilities [17–20]. Data from high- and middle-income countries demonstrate the consequences of foregone or delayed NCD-related healthcare seeking. These include poorer rates of diabetes diagnosis, control and up-titration of medications, and poorer CVD outcomes due to decreased access to care [21–24]. Reduced facility attendance or admission for acute NCD complications, such as heart attacks, often increase out-of-hospital deaths, and worsen long-term complications, including functional impairments and disability [20].

Some humanitarian actors have signalled their ability to continue NCD services with minimal disruptions during the peak of the COVID-19 pandemic [25], an ability that was not demonstrated even in stable high- and middle-income settings in the early phases of the response [18]. However, we know little about how COVID-19 disrupted NCD services in crisis settings more broadly, how actors adapted, and what factors enabled or hindered them to do so.

Though the peak of the COVID-19 pandemic is behind us, it is important that we learn lessons from this experience that may shape future NCD services and policies. Given the likelihood of another pandemic, and the fact that the climate crisis will cause more extreme weather events and compound the vulnerabilities that lead to conflict, WHO and other actors are placing greater emphasis on health system preparedness, response, and resilience. Therefore, factors affecting continuity of care for NCDs and successful adaptations to care delivery in the context of COVID-19 are important for preparing for future health service disruptions, for ongoing crises, and where marginalised or vulnerable communities have limited access to care [26]. Accordingly, we sought to explore factors affecting implementation of NCD care in crisis settings during the COVID-19 pandemic in LMICs, and the adaptations made to support implementation.

## Methods

### Study team and setting

The Centre for Global Chronic Conditions, in collaboration with the Health in Humanitarian Crises Centre, from the London School of Hygiene and Tropical Medicine (LSHTM), led the study in partnership with the Global Alliance for Chronic Disease (GACD) Humanitarian Crises Working Group. The research design was guided by an advisory committee of experts from key humanitarian organisations and agencies [WHO, United Nations High Commission for Refugees (UNHCR), International Committee of the Red Cross, Médecins sans Frontières, and International Rescue Committee] who work on global policies and programmes delivering NCD care in humanitarian settings. This was a global study, targeting humanitarian actors in all geographical settings, who were involved in direct delivery of NCD care during the COVID-19 pandemic.

### Study design

The study used a newly developed online survey in English (Additional file 1) targeting humanitarian actors, followed by individual interviews (Additional file 2) with selected participants. We focussed on the delivery of care for hypertension, type-1 and type-2 diabetes (“DM/HTN”, implying care for either or all conditions) as these are the most common NCD types currently addressed by humanitarian organisations [13, 27]. These conditions are also established tracer conditions, used in the healthcare quality assessment literature to assess health system or service performance [28–30]. These example conditions tend to be well defined, prevalent, relatively easy to diagnose, and have effective, available treatments.

### Conceptual framework and definitions

**Fig. 1:**
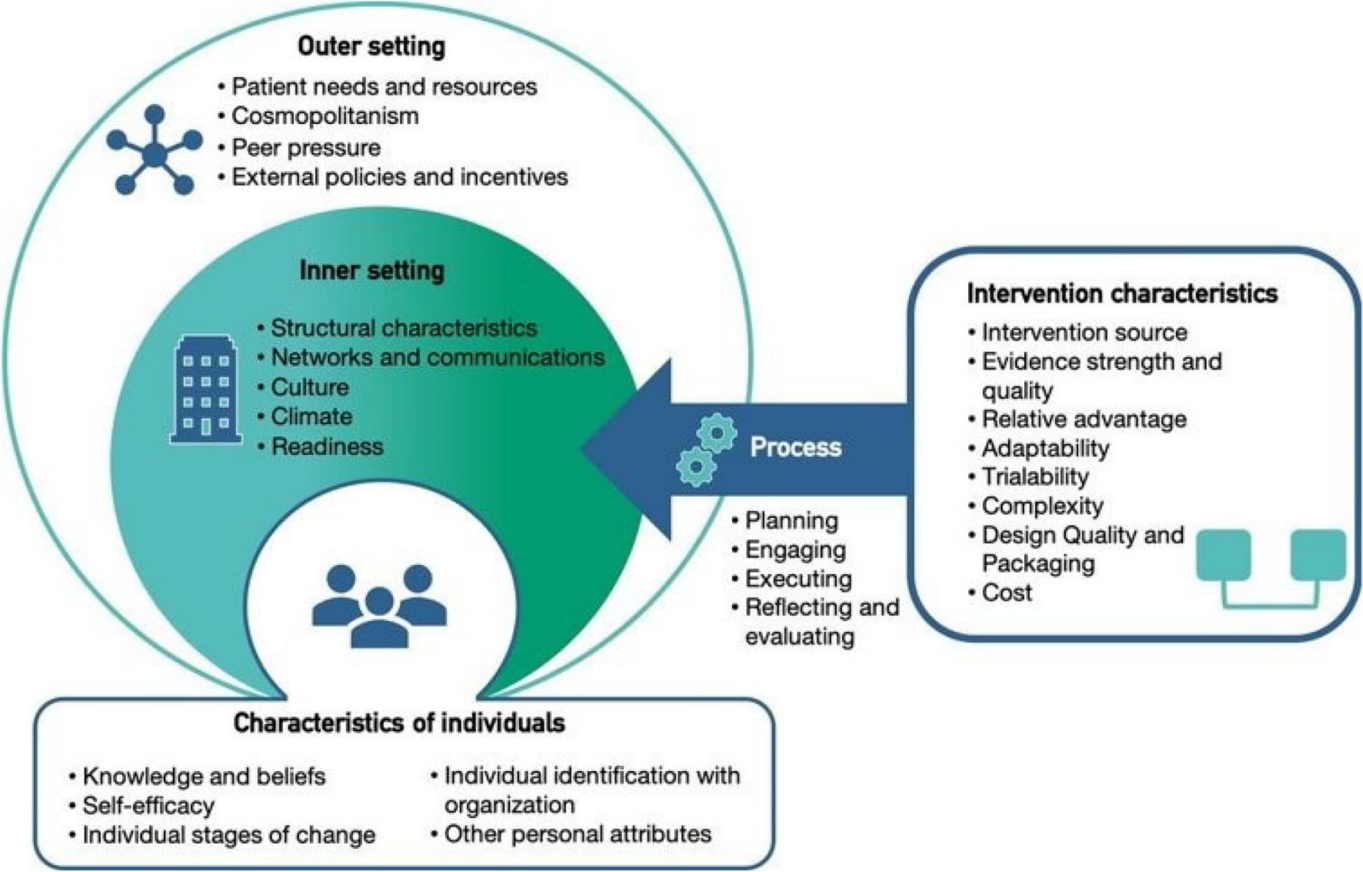
The Consolidated Framework for Implementation Research framework (2009), Source: [31, 32]

We used an implementation science framework, the Consolidated Framework for Implementation Research (CFIR – Fig. 1) to inform the design and analysis of the survey and interviews [31, 32]. CFIR is a practical framework, which provides a list of constructs, organised within domains, that are believed to influence implementation, either positively or negatively. It is intended to help guide the systematic assessment of potential barriers and facilitators and, thus, tailor implementation strategies and adaptations, and/or to explain outcomes. The five major domains of the framework – 1) intervention characteristics, 2) outer setting, 3) inner setting, 4) characteristics of individuals, and 5) process – provided a means to synthesise diverse interventions or adaptations in various contexts in response to a global pandemic.

For this study, we conceptualised the “intervention” as maintaining access to NCD care while responding to the health risks of the COVID-19 pandemic. “Maintaining access to care” was defined as the continued provision of care to the target population at a minimum acceptable level, compared to the baseline (e.g., before the pandemic), so that the services were available (i.e., with adequate human resources, equipment – including drugs – to safely deliver quality services), physically accessible and affordable, and utilised by the target population. NCD care refers to primary health care level activities for people with hypertension and/or diabetes that we propose are essential to maintain during the COVID-19 pandemic.

### Data collection

The online survey (Additional file 1) was designed by the LSHTM team, guided by the CFIR framework constructs, reviewed by the advisory committee, and piloted. Questions focussed on the delivery of a specific programme/project, focussing on the characteristics of pre-pandemic NCD services, adaptations made in response to the pandemic, individual and inner and outer setting challenges or facilitators, and decision making. We defined the components of NCD services as: medical consultation, disease monitoring, PLWNCDs’ education and support services, and primary prevention and community screening. The survey was hosted on the BOS Online Survey tool ©. A survey link was shared with all participants via email, and the survey included screening questions to restrict participation to people with relevant profiles. It was launched in March 2021 and closed in June 2021.

For the in-depth interviews, a structured topic guide (Additional file 2) was used to direct the flow of conversation, and ensure coherence of discussions with the study’s aims and survey. To facilitate rapid data collection, a team of four female interviewers with a public health background (CS, AC, JS, RI) was trained by EA. Each interviewer invited two to four participants and undertook between one and three interviews. From October to December 2021, thirty participants were contacted by email, of whom 13 took part in an interview. Interviewers probed the participants with follow-up questions based on their unique responses, and at the interviewer’s discretion. Interviews took place from November to 2021 to January 2022, and lasted between 45–60 min. They were conducted online, over the phone, or via Skype or Zoom audio-conferencing platforms. Interviews were conducted in English and were digitally audio-recorded, and transcribed for analysis using MS Word and Excel. Written, informed consent, was transmitted via e-mail. Weekly meetings were held with the study team to debrief on interviews, discuss initial findings and iteratively adapt the topic guide.

### Participant sampling

Project managers or medical staff directly involved in NCD care delivery at project/programme level in humanitarian settings during the COVID-19 pandemic were eligible for the online survey. Programming professionals are directly involved in the implementation of NCD programmes and service delivery, and their tacit working knowledge and experience provide invaluable insights into how the COVID-19 pandemic and policies affected NCD programmes, as well as how adaptations were formulated, coordinated, and implemented during this crisis. Using our existing GACD, LSHTM, and advisory committee networks, our partners emailed a convenience sample of their contacts who fit the sampling criteria, sharing information on the study, and inviting them to fill in the online survey. Snowball sampling of the respondents’ contacts was used to extend the sampling frame.

A sub-set of survey participants was invited to participate in in-depth interviews, six months after the survey was administered. The interview cohort was purposively selected to represent voices of participants in a range of roles in NCD programmes, from different organisation types that employed different types of adaptations, across different global regions. With input from the advisory committee, the study team defined the following selection criteria to identify follow-up interview participants: 1) geographical spread, 2) range of adaptations/ adjustments, 3) range of organizations, and 4) range of positions/ roles in NCD care delivery.

### Data analysis

Descriptive tabulation of quantitative survey responses was undertaken using the Stata statistical software package [33]. The survey was conducted as a rapid response to the initial phase of the pandemic, and early findings were shared with the advisory committee.

Qualitative data from a) survey free text responses, and from b) interview transcripts, were analysed jointly, using a combination of Framework Analysis (deductive coding) and inductive open coding approaches [34]. The Framework Method provides clear steps to follow and produces highly structured outputs of summarised data. It is therefore useful where multiple researchers are working on a project, particularly in multi-disciplinary research teams where not all members have experience of qualitative data analysis. First, an a priori coding template using MS Excel was developed by EA based on the CFIR framework (Fig. 1) to guide the deductive coding process (performed by OH, AC, CS). A separate data-driven inductive coding exercise was conducted by EA and LV. Repeated review and the complimentary coding approaches enriched the research team’s interpretive and analytic understanding of the data. The qualitative data is presented as reconstructed narratives using both a descriptive and interpretive stance, by themes, and with direct quotes from the participants.

## Results

The survey received 98 responses, from 38 different organisations, operating in 21 different countries. Most survey respondents were working in South-East Asia, Africa, and the Eastern Mediterranean (34%, 33% and 28% respectively), and their programmes were based in protracted conflict areas (32%), and targeted refugees (83%), although 60% targeted mixed populations [i.e., a mix of refugees, internally displaced populations (IDPs), and/or host populations]. Most programmes were in camp settings (70%), and provided DM/HTN care integrated within general primary health care (63%) or with other NCDs (including cardiovascular disease and mental health care) (26%). Table 1 outlines the characteristics of the survey respondents and the NCD programmes they were involved in.

**Table 1:** NCD programme characteristics among survey respondents (*n* = 98)

NCD Programme Characteristics	Frequency	Percentage
**Region**		
Africa	33	33
Eastern Mediterranean	28	28
Americas	1	1
Europe	1	1
South-East Asia	34	34
Western Pacific	1	1
**Humanitarian context**		
Natural disaster	5	5
Acute conflict	12	12
Protracted conflict	32	32
Public health emergency	24	24
Don’t know	1	1
Other	2	3
**Programme target population**		
Refugees	81	82
Internally displaced population	13	13
Returnees	13	13
Host population	54	55
Other	9	9
Mixed population (more than one population)	60	61
**Programme location**		
Urban/peri-urban	35	35
Rural	32	32
Camp	71	72
Non-camp	20	20
Other	2	2
Mixed location (more than one location)	40	40

Interviews were conducted with 13 of these survey respondents. Table 2 outlines the interview participants’ characteristics.

**Table 2:** Interview participant and NCD programme characteristics (*n* = 13)

Item	Characteristics	Number of interviewees
**Region**	Middle East and North Africa	5 (Syria, Iraq, Lebanon, Jordan)
	Sub-Saharan Africa	5 (Rwanda, Uganda, Kenya)
	Asia	3 (Bangladesh and Thailand)
**Location**	Camp settlement	8
	Urban/Peri-urban (and rural)	4
	Rural non-camp	1
**Target population**	Refugee population	5
	Host population	1
	Both	5
	Unknown	1
**Interviewee role**	Clinician	5
	Team Lead	5
	Other (Executive, Community Health Worker, Research)	3

Findings from both the survey and interviews are reported below, following the CFIR implementation framework constructs (*intervention characteristics, process, outer setting, inner setting,* and *characteristics of individuals*) and subconstructs, which are highlighted in italics. As mentioned, we defined the “intervention” as maintaining continuity of NCD services, while mitigating the threat of COVID-19.

### Intervention characteristics

Before the pandemic, medical consultation was provided by generalist doctors in 90% of respondents’ NCD programmes; specialist doctors, nurses, and lay- or community-based health workers/volunteers were involved in 27%, 41% and 43% of respondent’s programmes, respectively. Consultations were done individually and face-to-face in most (98%) cases. Groups were utilised for consultation and monitoring, but mainly for education and prevention/screening activities. Most medical consultations were delivered in a primary care centre or health posts (89%), fewer in secondary or tertiary level hospitals (36%), and services included home visits in 25% and mobile clinics in 15% of cases.

During the pandemic response, more than half of the NCD service components provided before the pandemic were partially or fully maintained, including medical consultation (94%), disease monitoring (90%), PLWNCDs’ education and support (88%) and primary prevention and community screening services (61%). As might be expected, face-to-face individual services declined, with more than 50% of these services reduced during the pandemic, and medical consultation via home visits were cut by half. More detail on the characteristics of NCD service components before and during the pandemic are available in Additional file 3.

Organisations’ implementation processes varied as they experienced different organisational (*inner setting*) and contextual (*outer setting*) barriers and facilitators. Services were adapted iteratively as the pandemic progressed. For example, survey respondents reported *outer setting* factors that hampered continuity of service delivery, including poor mobile phone coverage (28%), smartphone availability (35%) and internet connectivity (35%). PLWNCDs faced challenges in managing their disease, especially financially (49%) and mentally (42%).

The key CFIR *intervention* constructs that were generated from interview and survey free text data were *source, evidence strength and quality, adaptability,* and *cost.* At the onset of the pandemic, national policies immediately targeted infection prevention and control (IPC) to limit the pandemic’s spread, introducing movement restrictions, and diverting health system policy and resources to the pandemic response. In the early days, interviewees reported initial uncertainty in how to respond to these policies.

The decision to prioritise PLWNCDs and the specific adaptations made to service delivery were perceived as coming strongly from within individual organisations, with recommendations coming from WHO/UNHCR, rather than from national governments. The latter were largely perceived as having “*forgotten*” PLWNCDs in their initial pandemic response plans. The *source* of IPC guidance, training and equipment was perceived to be national governments, Ministries of Health, and international actors, such as the WHO and UNHCR. The UN sources were considered trustworthy and of good quality, filling essential gaps when information or action was lagging from national resources. The *cost* of maintaining NCD care was mainly spoken of in terms of the cost and diversion of funds into IPC measures, and the fact that pandemic-related inflation increased costs for governments, organisations, and PLWNCDs, for example, significantly increasing transportation costs. The CFIR constructs *complexity, trialability* and *relative advantage versus other interventions* did not feature strongly in the data. There were many unknowns at the beginning of the pandemic response, and there was acknowledgement that organisations did not have time to trial interventions but, instead, needed to act quickly.

### Process

In most settings, the process of maintaining NCD care could be summarised as involving the following key components: a) the introduction or enhancement of IPC measures; b) prioritisation of PLWNCDs and maintenance of clinical contact, including through remote means; c) maintenance of medication and equipment supplies; d) maintenance or adaptation of the health workforce; e) information sharing between organisations and with PLWNCDs, and countering misinformation; and iteratively adapting these approaches as the pandemic evolved:

*“Adaptations done in NCD service delivery were aimed to address the safety of NCD patients from COVID-19, considering their susceptibility to mortality due to COVID-19, also safety of health care staff, from community level to health facility level” [ID01]*

The CFIR constructs *planning, engaging, executing,* and *reviewing* were discussed in interviews and survey free text responses. *Evaluating* was less prominent in the data, given that data were collected relatively early in the pandemic response, and programmes did not have time to formally evaluate their response strategies. However, respondents reported anecdotally that their interventions were successful.

The WHO Health Sector Cluster System or UNHCR-coordination systems, which are used to coordinate multiple agencies during emergency responses, were instrumental in planning and executing the pandemic response in places where it was already established. For example, in these settings, collaboration and information sharing occurred early in the pandemic. Decisions on how to respond were generally made by the organisation’s management, although one interviewee described close engagement of clinical staff in an iterative decision-making process:

*“…clinic staff, budget staff and … coordination, all three … were working together to come up with these recommendations of how to overcome the challenges at the clinic level. So, I think the recommendations came mostly from the clinic staff …but it was a collective decision. [ID31]*

#### Infection prevention and control

Interview participants described rapidly introducing COVID-19 risk mitigation measures, including IPC protocols, such as the use of personal protective equipment (PPE), hand hygiene, and social distancing, and training on the clinical management of COVID-19. A number of participants noted there were supply delays in some circumstances. Where organisations initially suspended DM/HTN services, shortages in PPE (14%) was the most commonly reported reason. Masks and PPE were introduced as soon as supplies were available and were often provided by international non-governmental organisations (NGOs) and United Nations (UN) organisations, who stepped in when national supply chains were inadequate or too slow.

#### Prioritisation of people with NCDs

Respondents consistently reported that their organisations, unlike many national governments, recognised the increased risk PLWNCDS faced, and the need to prioritise their continuity of care. Organisations took varying approaches to social distancing to protect and prioritise PLWNCD and staff. For example, in some contexts, outdoor waiting areas were created, and temperature checks and triage of PLWNCDs were introduced. PLWNCDs were often separated from other primary care patients. In many, although not all, cases, only PLWNCDs with severe or uncontrolled conditions continued to be seen at facilities, by appointment only, while those with stable conditions were advised to remain at home. In a minority of cases, facility-based consultations were maintained for all PLWNCDs, while group-based activities were adapted (Additional file 3).

#### Maintaining NCD consultations

Table 3 outlines the survey response on the change or termination of NCD programmes implemented by the respondents’ organisations. Medical consultations were largely maintained or immediately adapted – only 12% of respondents reported initially suspending and then resuming them in an adapted format. The major reasons reported for suspending consultations were government-mandated movement restrictions (33%) and PLWNCDs’ fear of face-to-face attendance (24%). These factors also reduced the numbers of consultations in the initial months.

**Table 3:** Change in NCD programme components’ delivery during pandemic

**Service**	**Medical consultation (%)**	**Disease monitoring (%)**	**PLWNCDs education/support services (%)**	**Primary prevention/ community screening (%)**
Unchanged	33	46	35	22
Adapted	55	37	38	48
Suspended temporarily (and unchanged when resumed)	0	8	11	7
Suspended temporarily (and adapted when resumed)	12	10	14	16
Completely stopped	0	0	2	6

Other NCD programme components were also adapted, either immediately or after a period of brief suspension. In most cases, disease monitoring continued unchanged (46%), and the remainder of programmes simplified or reduced monitoring frequency. The few service components that were completely stopped without resumption tended to be at the community level (2% of education and support services, and 6% of primary prevention and community screening services) or involving group-based activities or mobile units (Table 3 and Additional file 3).

#### Reducing facility-based contact

Adaptations were introduced to maintain contact when PLWNCDs could not attend facilities. Face-to-face consultations were either dropped entirely (reducing from 93 to 39%) or decreased in frequency (73%). The principle means used to maintain contact with PLWNCDs remotely were via community health workers or volunteers (CHW), and via use of telemedicine.

CHWs were involved in some aspect of NCD service provision, mainly in education and support and/or NCD prevention and screening activities (Additional file 3). They played a role in medical and in disease monitoring in about one fifth and one third of cases, respectively. In response to the pandemic, one fifth of respondents (21%) reported additional task sharing to community-based staff. Their role was expanded to include education around COVID-19, IPC, and vaccination, active follow up of PLWNCDs, home-based clinical and adherence monitoring, and liaison with clinicians, supporting remote management of PLWNCDs. Interview participants from diverse settings highlighted the key role that CHWs played in reaching the community and gaining real-time insights on community needs, disseminating information, and gaining community trust.

In parallel, however, participants emphasised the need for adequate and regularly updated training, communication pathways, and support for CHWs:

*“We ensured CHWs (were) kept on their toes in terms of trainings and refresher, information on COVID and NCD and management of NCD within the COVID-19 pandemic. Two, we ensured that CHWs also (were) giving (clinical) information back …It’s also very important to have (a) communication system where CHWs can … share information directly to you and … tell you the situation in the community…. [ID26]*

**Fig. 2:**
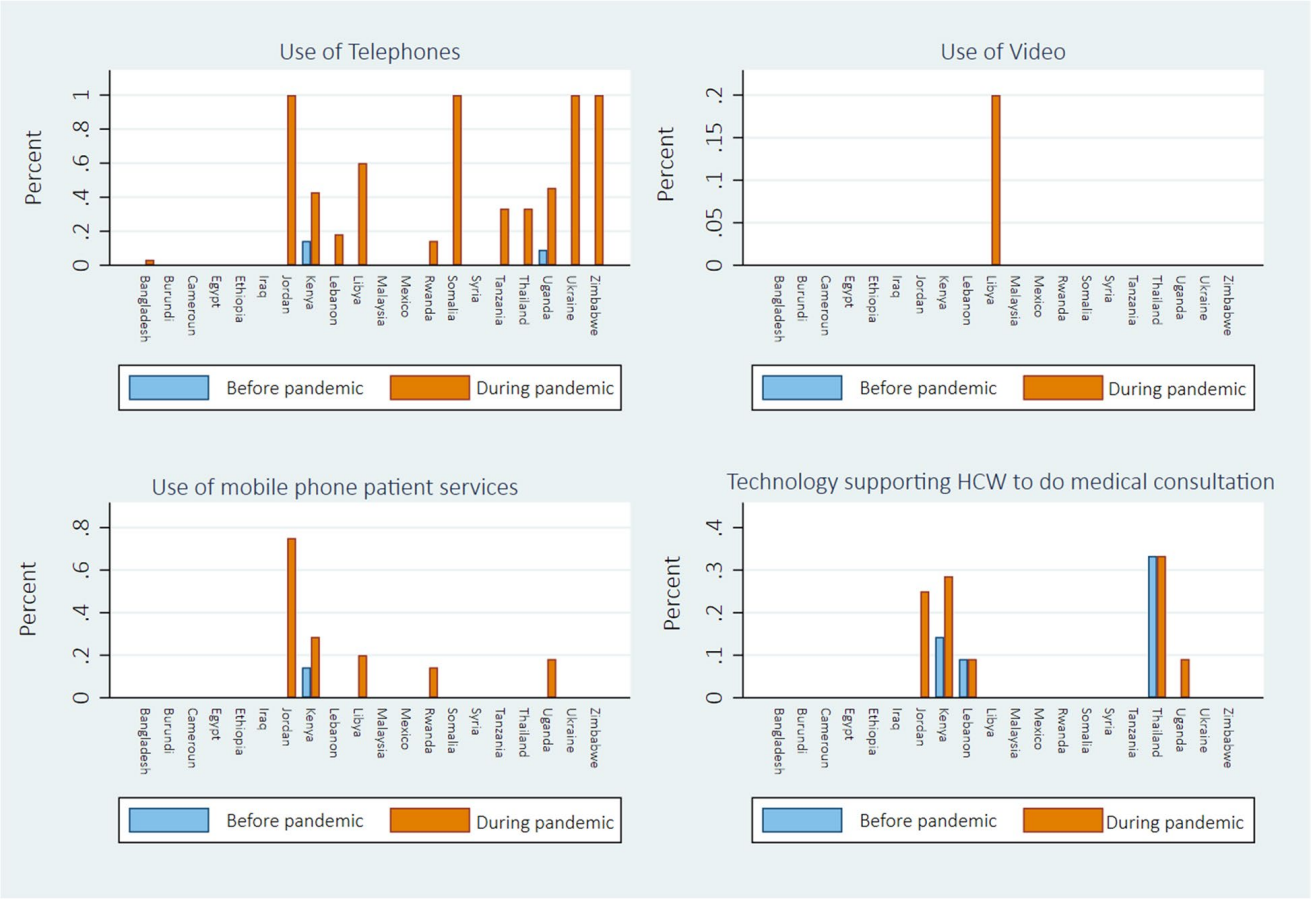
Use of technology to support medical consultations before and during the pandemic

Prior to the pandemic, the survey findings suggest that telemedicine via mobile or landline telephone, WhatsApp, or video consultation, was utilized by a very small proportion of our study respondents’ organisations (Additional file 3). The survey results also indicate a higher use of telephones during the pandemic to provide medical consultations, disease monitoring, education and support services, and primary prevention and screening. For example, 2% of respondents reported their organisations using telephone consultations pre-pandemic, which increased to 23% during the pandemic (Fig. 2).

Access to and use of blood pressure and blood sugar monitors was variable. Similarly, access to digital devices with internet connectivity such as telephone, smartphones, and tablets, to communicate remotely with health facilities varied significantly. Where there was phone and internet connectivity and access to use of smart devices, programme staff were able to engage with, and monitor PLWNCDs through online platforms. Stable PLWNCDs with controlled disease were supported to self-manage at home via phone consultations or CHW visits, and this was facilitated by PLWNCDs having home monitoring devices (blood pressure machines and glucometers). This was more common in the Middle East and North African region than in Sub Saharan Africa. Lack of available self-care resources in other settings meant that PLWNCDs were not able to monitor and manage their health within their homes. In one setting, PLWNCDs were taught to self-inject insulin rather than having to attend the facility for health workers do it.

In some instances, this change in remote consultation approach was met with initial resistance. As the approach was normalised, PLWNCDs reportedly began to prefer these modes of communication.

Communication via these platforms spanned from health education and awareness, to targeted counselling and psycho-social support, where its wide reach was deemed beneficial in reducing stigma. For example, one programme provided nurse-led psychosocial support via WhatsApp groups. Uptake was increased through the delivery of “*ice breaking*” messages and the service was offered to all PLWNCDs, and therefore engagement with the service was not associated with having a mental illness.

Several examples of the CFIR constructs *reviewing and evaluating* were offered by interviewees. For example, several organisations realised that their initial attempts to use internet or smart phone-based technology were hampered by PLWNCDs’ lack of or uneven access to digital infrastructure, and they reverted to using telephones or community health workers to maintain contact. One interview respondent also described realising, after a period of implementing phone consultations, that doctors required specific guidance and tools to undertake these safely and consistently.

#### Maintaining supply of medication and equipment

At the beginning of the pandemic, most interview participants described issues with procurement of medication and IPC equipment, and national level supply chains being diverted to the pandemic response. Supply issues were reported as the main reason some programmes initially stopped or suspended DM/HTN service. In addition, almost half (45%) reported internal supply issues within their organisation which hampered continuity of care, and one third (32%) reported introducing adaptations to medication procurement or supply in response to the pandemic.

Key adaptations to medication supply included increasing the dispensing interval to three months (32%) (following WHO guidance), allowing family and friends to pick up medications from facilities (48%), and in one case, having community health workers deliver medication to people’s homes. The reduced frequency of medication pick-ups was seen as a useful to mitigate exposure to the virus in high-risk populations and to reduce crowding, caseload, and the number of people in health facilities.

Interviewees indicated that supply chain challenges lasted up to about four months and were resolved through national and international interagency collaboration.

#### Maintaining the health workforce

Survey participants cited staff absence due to COVID-19-related illness or quarantine (60%), and staff burnout (49%) as key internal organisational challenges to maintaining continuity of NCD care during the pandemic. Many health care workers were diverted from their usual roles to the pandemic response, their movements were physically restricted during the “lockdowns”, and interviewees recounted their initial “*panic”* and high stress levels.

Strong interorganisational collaboration, particularly within camp settings, allowed organisations to pool their human resources and “cross-cover”, for example, taking on another organisation’s PLWNCDs when they had a COVID-19 outbreak among staff. One organisation reported creating two teams of staff who worked in separate shifts, to minimise burn-out and infection risk. To alleviate these workforce challenges, several reported task-sharing within the facility (25%) and/ or to community-based staff (21%) (Additional file 4). Interviewees cited improved supply of PPE and the introduction of COVID-19 vaccines as pivotal changes that protected staff and reduced their fear.

#### Sharing information and countering misinformation

Themes around use of existing data and data sharing between organisations were generated inductively from the interviews. The importance of patient registries was clearly highlighted, since they allowed staff to track NCD patients, which enabled continuity of care, and information sharing with patients. Where the WHO Health Cluster and UNHCR coordination mechanisms were strong, particularly in camp-based settings, agencies pooled their NCD patient lists and supply data, allowing agencies to share resources and collectively respond.

Communication strategies were key throughout the pandemic response. During the initial phase of the pandemic, programmes focussed on urgently communicating the infection risks and prevention strategies, through public and programme-based communication. Additional messaging on the importance of follow-up care for NCDs was then necessary, to counter people’s fear of attending facilities. Once vaccines were introduced, a new wave of messaging was required and implemented in many of the programmes—this time on the merits and safety of COVID-19 vaccines, and to counter misinformation and vaccine myths.

*“At the beginning it was very difficult. You know, the misinformation “oh the COVID-19 vaccine it makes you die.” …we worked in coordination with other health services with the refugee camp and community health volunteers conducting home visits to ensure all NCD patients (got) the vaccine… [ID09]*

Community health workers, where they were active, played an important role in delivering these messages, and interviewees also reported using social media, such as Facebook and WhatsApp, SMS messages in some settings, and more traditional loudhailers to spread educational messages, where settings were conducive to this e.g. in camps.

### Inner setting

*Structural characteristics* of the surveyed organisations – most of which were humanitarian actors used to working in volatile settings, assessing acute needs, and rapidly intervening – and their internal *networks and communications* were important elements in quickly responding, and iteratively adapting to the pandemic. Narratives from the interviews, which were conducted about six months after the survey took place, highlighted that after the initial uncertainty, programme staff felt better equipped to manage the evolving circumstances. Interviewees highlighted their organisations’ resilience, inherent agility, and ability to adapt, and several expressed pride in their organisation’s success in coping, and maintaining continuity of care for PLWNCDs. Furthermore, teamwork and coordination were often strengthened by the pandemic response and several respondents proposed retaining these adaptations after the pandemic.

The physical infrastructure and camp versus urban setting characteristics were highly influential. Movement within camps was less challenging than moving in and out of camps, or within urban areas, and, where host populations used health services within camps, their access was jeopardised.

Strong baseline data collection systems and processes within an organisation enabled assessment of the situation, follow-up of individual patients and data sharing with other organisations:

*“In our facility, we have one dedicated register for non-communicable diseases patient… so our dedicated team, continuously (kept) tracking these patients…and we (kept) connection with our community health workers ...” [ID02]*

However, other organisations felt hindered by the lack of available data and data infrastructure in planning and rolling out their response.

Generally, interviewees were receptive to the changes that had to be made in response to COVID-19, the idea of protecting PLWNCDs, while maintaining continuity of care fit with individual and organisational norms and values. Interviewees generally felt they had support and feedback from managers. However, many described undertaking additional tasks with a reduced workforce and staff burnout as a prominent theme in both survey and interviews. Some participants also described a lack of “back-up” emergency plans, including alternative workflow plans when staffing was short.

Views on PPE training were mixed; some described it as delayed or improperly carried out. There were also contrasting accounts of CHW training, which was poor in some settings and highly successful with bespoke CHW training packages being developed in other settings. Overall, quick development and dissemination of training programmes, including for non-medical and CHWs, often through online/remote modules from various international and local health actors were recognised as an important enabling factor in continuing NCD care in a safe manner:

*“All health workers had training about the IPC measures during COVID-19, and how to deal with patients. This was online training… done at the beginning of the crisis, through the WHO…on their website….” [ID09]*

### Outer setting

Participants were asked about their awareness of *PLWNCDs’ needs and resources* and their attempts to prioritise them. Survey respondents cited physical restrictions (88%), social restrictions (60%), fear of attending health services (54%), financial hardship (49%), and poor mental health (42%) as the key challenges faced by PLWNCDs during the pandemic (Additional file 4). They attempted to overcome them by introducing remote modalities for consultations and monitoring, and strong, agile messaging campaigns.

As anticipated, respondents highlighted established structural and infrastructural challenges in providing NCD care that existed before the pandemic, including a lack of NCD policy and funding and national economic pressures. More general challenges faced by humanitarians operating within an emergency response, such as fragmented health systems, with pluralistic actors, sometimes operating in vertical programmes with limited integration, were also noted.

The degree to which an organisation was networked with other external organisations (*cosmopolitanism* within CFIR) proved a crucial enabler in rapidly adapting and maintaining care for PLWNCDs during the pandemic, and a key theme that was identified from surveys and interview data. Interviewees described utilising pre-existing networks of health actors and WHO-led health cluster meetings, especially in camp-based settings, with a significant strengthening of these relationships, and day-to-day collaboration increasing far beyond pre-pandemic levels. Examples of this included creating a master list of NCD patients within camps, cross covering each other’s operations and borrowing each other’s resources, including health workers, medical supplies, and community volunteer networks. These networks offered key support and a degree of *peer pressure* or competitive pressure to implement interventions.

One example of a new cross-sectoral collaboration was offered, whereby a health organisation repurposed a CHW network, which was usually involved in protection activities, to engage in active follow up of PLWNCDs. Government stewardship and leadership were also highlighted as key enablers to rapid response and adaptation.

*External policies and incentives* played a key role as either barriers or enablers. The lack of national-level emergency preparedness plans and mechanisms for coordination between health actors were highlighted by many respondents. Narratives around the early instructions from various Ministries of Health suggest a strong initial focus on infection control, and de-prioritisation of other services, including those for chronic disease:

*"COVID took all the, let's say the light and only cases with COVID were prioritized. So no, I think NCDs were pulled back during the pandemic." [ID09]*

A lack of pre-existing national-level policies and funding for NCDs, followed by the diversion of funding and staff time in public facilities to infection control measures and COVID-19 treatment hampered the continuity of NCD services and referrals. External policies by partner hospitals or health facilities also influenced the continuity of some NCD programme components. For example, non-emergency referrals to secondary and tertiary care hospitals were often postponed.

Other potential adaptations to reduce facility-based contact for PLWNCDs were hindered by the lack of enabling policies and national infrastructure. For example, policy barriers prevented longer-term dispensing of medicines in some contexts, and the lack of legal mechanisms to enable task sharing or telehealth consultations limited adaptions of service delivery in others. The baseline utility and availability of technology in the local context was a clear influence on the remote care modalities that could be introduced. Respondents reported a lack of national infrastructure to facilitate virtual or remote health activities prior to the pandemic, including for consultations, prescriptions, and medication delivery. Thus, while organisations were initially advised to use social media, smartphones etc., many found that this was unrealistic in their settings.

Persistent advocacy and engagement with Ministries of Health was successful in changing the policy approach towards NCD services and dispensing of medicines. Respondents suggested further advocacy was needed with governments to include NCDs as priority conditions in future emergency response, to allow for longer dispensing intervals to reduce the burden of facility attendance, and to build on technology and infrastructure to allow for remote consultation and dispensing. In Table 4, below, we summarise our findings around the contextual factors, intervention characteristics and other barriers and enablers that influenced the continuity of NCD care in humanitarian settings during the COVID-19 pandemic. We also note our study participants' recommendations for action to maintain NCD care continuity during future crises (Table 4).

**Table 4:** Challenges and enabling factors to maintaining continuity of NCD care in humanitarian settings during the COVID-19 pandemic, adaptations made, and recommendations for future

Inner and outer settings	Encountered challenges, constraints & barriers to continued service delivery	Intervention characteristics and adaptations	Enabling factors to implement change and adaptations for continued service delivery	Participant recommendations for future action
**National government policy landscape, and health actor partnerships**	• Early de-prioritisation of NCD services at policy level • Policy barriers that prevented longer-term dispensing of medicines • No legal mechanism to enable telehealth consultations • No/lack of emergency preparedness, including mechanisms for health actor coordination • Lack of national virtual/remote health activities and infrastructure prior to pandemic-including consultations, prescriptions, deliveries	• Policy reversals of NCD de-prioritisation and limited dispensing • Close collaboration between agencies involved in NCD care, including within camps and sharing of resources such as staff, medicines, etc	• Government stewardship and leadership in coordination • Advocacy to prioritise NCD services in health systems, and during future crises • Pre-existing multi-year programmes provided enabling dynamics and platforms to implement a range of responses	• Strong long-term coordination and collaboration between actors and strengthened referral mechanisms • National policies that enable longer term dispensing for stable PLWNCDs, which can reduce crowding and burden at facilities
**National and programme health financing**	• Limited pre-pandemic health and NCD budget and currency inflation • Diversion of financial resources for NCDs to COVID-19 IPC			• Dedicated and protected budgets for NCDs, including in emergency and crisis plans
**Facility/ programme level: service delivery & infrastructure**	• Increased dependence on digital technology without adequate internet, phone and hardware (smart phones, tablets etc.) for staff and PLWNCDs • Frequent temporary closure of whole facility for disinfection • Difficult to implement social distancing guidelines due to lack of necessary space/ venues	• Starting PLWNCDs self-management during pandemic • Using outdoor spaces for consultations, use of personal protective equipment, improved hygiene, social distancing • Pre-entry triage and temperature monitoring to screen needs and risk- prioritisation for PLWNCDs • The creation, use and tracking of PLWNCDs’ outcomes database or spreadsheet to aid follow up and management • Enforcing appointment-based attendance, and not allowing walk-ins • Reduced monitoring frequency of stable PLWNCDs to yearly • Installation of plexiglass in reception, pharmacy and consultation rooms • NCD app development to help with follow up and use of asynchronous platform for remote consultations • Reduced number of PLWNCDs daily • Use of Facebook Messenger and WhatsApp as digital platforms for consultations		• Digitalisation of data system related to NCD service delivery should be introduced in NCD operations of all organisations
**Facility/programme level: Access and supply: medicines, digital health, PPE & diagnostics**	• Insufficient personal protective equipment • Procurement limited by unduly long bureaucratic processes • Medicines and medical resource use consumption patterns changed, and visibility of change and stock affected • Disrupted continuity of care even for severe presentations of NCDs, and simplified diagnostics based on WHO PEN protocol	• Longer-term dispensing for stable PLWNCDs (i.e. from dispensing bi-weekly or monthly to dispensing two–three months’ supply) • Enable family/friends’ prescription pick-up	• Available stock and enabling policy shifts to enable longer-term dispensing	• Increase stockpile of drugs and longer-term dispensing • Long-term programme to enable family/friends’ prescription pick-up
**Facility/programme level: Human resources for health**	• Initial staff panic • Increased stress levels • Lack of, or unfamiliarity with back-up plan in change of service workflow • Health worker shortage due to illness, quarantine/ isolation • Inadequate and delayed capacity-building • Community health worker training and programmes ineffectively implemented	• Scale up of community health worker/volunteer (CHW) role • Psychological and mental health support • Training on IPC and on clinical management of SARS-COV-2 infections • Division into two or multiple teams to mitigate loss of workforce from virus exposure • Engaging CHWs for education, managing fear and misinformation, monitoring, engagement with non-attenders and delivering medication	• Pandemic provided the opportunity to scale up the work of CHWs that had started but was prioritised during the pandemic • Systems and plan for flexible adaptations • Early development and dissemination of guidance	• Staff incentive and engagement programme • IPC training for CHWs • Use of online training • Implement and disseminate SOP and training on change management/normalisation • Scale-up and strengthen the linkage between community health programme and NCDs
**PLWNCDs factors: health-seeking, community, and household resources**	• Beneficiaries not tech-literate • Increased dropouts or insufficient follow-up of PLWNCDs due to reduction in appointment frequency, less disease control • Lack of home-monitoring equipment	• Provide emotional support to PLWNCDs • Starting PLWNCDs self-management during pandemic; stable PLWNCDs managed in home settings • Distribution of cloth face masks	• Utilising existing relationships of trust, through community leaders, volunteers, etc	• Long-term home-based care plan, access to self-care resources and social support- volunteer/ CHW visits for NCDs management • Inclusive outreach and long-term engagement with local communities

## Discussion

To our knowledge, this is one of the first studies to document factors affecting the implementation of NCD care in LMIC humanitarian settings during the COVID-19 pandemic [25]. A key finding was that NCD services were largely maintained throughout the pandemic response. Respondents’ organisations minimised interruptions to NCD care, while mitigating the risks of COVID-19, by adapting to enable remote care and reduce facility-based contact. Our study respondents highlighted how the pandemic response exacerbated the pre-existing challenges they faced in delivering NCD care in crisis-affected countries. Most humanitarian actors operate in fragile LMIC settings, where health systems are often under-resourced and fragmented, and where national-level emergency preparedness and response mechanisms may be limited. Reflecting the experience in other parts of the world, our data highlighted that initial COVID-19 responses seemed to de-prioritise PLWNCDs, health system resources were diverted away from NCD care and, especially in many LMIC settings, access to pandemic mitigation strategies, PPE and vaccines was frequently delayed [11]. Maintaining NCD care during the pandemic was also hampered by the lack of pre-existing policy or infrastructure to support remote care modalities, the fear and misinformation around COVID-19, and the initial resistance to remote care expressed by PLWNCDs.

Despite the challenges, humanitarian actors were adept at implementing context-adapted changes to support continuity of NCD services, which is consistent with findings from a similar study [25]. The humanitarian system’s in-built flexibility and agility, existing humanitarian coordination mechanisms, and strong experience communicating with PLWNCDs and advocating with authorities were all supportive factors. The UN agency coordination mechanisms, including the WHO health cluster approach and UNCHR working groups enabled quick coordination and sharing or repurposing of partner resources. When it was available, strong data collection on NCDs, such as patient registries and supply monitoring, underpinned this effective interagency coordination. Humanitarians’ experience with previous outbreaks, such as cholera and Ebola, while different, may have allowed them to react in a more agile manner than national health systems could. In keeping with this, LMIC countries that were most successful in their pandemic response built on prior outbreak experience and on existing community resources, including community health workers [14].

The key role of community health workers and volunteers in facilitating continuity of NCD care, sharing key information, and building trust among communities stood out in our data. This is consistent with other studies, which found that, with adequate and timely resources, including adapted protocols, training, and PPE, pre-existing CHW programmes were able to continue with minimal disruption during the pandemic [15, 33]**.** The key part CHWs played in many of the pandemic responses recounted here reflects their pre-existing role in refugee camp settings and within Sub-Saharan African and in Southeast Asian health systems. By contrast, the role is not often utilised in the Middle East and North Africa, and it has been highlighted as a potential area for development [35]. There is growing evidence for the positive impact of CHWs on NCD management both in stable LMIC settings, and in maintaining services during periods of disruption [36–41]. However, in expanding this role in future NCD programmes, lessons must be learned around the need to adequately support CHWs with resources, supervision and training [42].

Telehealth, defined as “the combined use of the internet and information technology for clinical and organisational purposes, both locally and remotely”, has been touted as one innovative approach to maintaining continuity of care for PLWNCDs that should be retained and built upon post-pandemic [43, 44]. According to the WHO, telemedicine and patient triage were the most common mitigation strategies used to reduce NCD service disruption in the early days of the pandemic [17]. However, our study reflects the literature around the introduction of telehealth – its success is highly contingent on national infrastructure, smartphone ownership rates, and internal organisational factors. Moreover, clear guidance, training and culturally-congruent communication all support its successful implementation [45]. Our data also highlight the need for guidance for clinicians in the use of telemedicine, in keeping with previous calls for specific WHO guidance on the development and use of digital health solutions for NCD care [20]. Narratives from this study suggest that the wider use of self-care, via home-based monitoring equipment, coupled with tele-health or CHW networks may be beneficial. These modalities may increase access to care, especially in crisis settings, where populations may be cut off from facilities, or where populations are marginalised or hard to reach. However, their cost effectiveness, acceptability and feasibility in different contexts must be tested with robust implementation research [46, 47].

Introducing telemedicine may increase health inequalities [42]. Throughout the pandemic, the use of digital health for NCDs has not been equitable across world regions, disease types, or populations [43]. Indeed, the COVID-19 pandemic has highlighted and entrenched existing global inequalities - essential health workers, migrants, refugees and other displaced or marginalised populations, and those living with NCDs were among the groups most burdened by its effects [14]. It shone a spotlight on the global NCD epidemic and the enormous negative health, social and financial effects NCDs bring, the magnitude of which far outweighs that of the pandemic [48].

### Implications and recommendations for practice and policy

Humanitarian actors and health systems continue to learn lessons from the COVID-19 response that may enhance models of NCD care. Our data support calls for more person-centred, community-based care that limits facility-based contact. Developing such models would be useful beyond the pandemic, as they bring care closer to people’s homes and communities and improve access by decreasing transport and time cost burden on vulnerable, resource-limited, and marginalised patients. They also decrease the risk of nosocomial infections, and potentially decrease the burden on health facilities and staff, allowing more time to be spent on quality care. The means of achieving this must be adapted to the context, but may include increased use of community health workers, telephone consultations, home-based disease monitoring and adapted dispensing practices. The potential for social media and CHW networks to spread reliable health messaging was also highlighted in our study. We recommend that new or adapted models of care should be co-developed with PLWNCDs, and evaluated for cost-effectiveness, using implementation research approaches. Training on NCDs and adequate supervision and funding is needed for health care providers – including CHWs – to build and retain their role in supporting communities. Increased funding and advocacy for the inclusion of NCDs in emergency preparedness and response is essential. Finally, we recommend further implementation research to evaluate some of the adaptations described here, for example, CHW- an/or tele-health supported self-care.

The COVID-19 pandemic exposed how underprepared the health systems of many countries were to respond to the global NCD epidemic. For example, only 42% of low-income countries included the continuity of NCD services in their national COVID-19 plan [20]. WHO has highlighted steps to “build back better” NCD services post-pandemic, such as including NCDs in national emergency response and preparedness plans, and strengthening baseline NCD data collection and NCD supply management systems [49]. In keeping with the “health for all” paradigm, NCDs should be integrated into strengthened primary health care within a universal health care approach, and access must be extended to people who are forcibly displaced by humanitarian crises.

### Strengths and limitations

This study was designed in the early days of the pandemic to gain insights that could be useful to humanitarians as they rolled out their responses. Engagement with an expert advisory committee, and pre-existing relationships with global humanitarian actors, provided access to respondents from multiple global regions. The survey and interviews took place at different time points in the pandemic, enabling the generation of insights relating to different response phases. Analysis was guided by an implementation study framework, which helped synthesise findings from diverse contexts.

However, the survey was not designed to identify the number of unique programmes, nor was it designed to detect differences in service delivery approaches before and during pandemic with statistical power. We cannot comment on the actual level of service use, on how it may have changed, nor on what impact any of the documented adaptations may have had on clinical outcomes, including complication rates and mortality.

We note that our survey’s initial convenience sampling approach, via study partners and existing networks, facilitated reaching major international humanitarian actors, such as UNHCR, but resulted in few local NGOs being included. This sampling frame meant that most survey participants worked in camp settings, despite most refugees and other forcibly displaced populations now living in urban, integrated settings [50]. The findings around enhanced communication and collaboration may, therefore, be less generalisable to non-camp-based settings. Despite producing a version in Spanish to encourage responses from South America, we had few responses from the Americas and from the Western Pacific. This was presumably because the major relevant NGOs had limited operations in these regions. Offering the survey in French and Arabic, for example, may have increased responses from other regions. Fewer than half of the invited interviewees accepted to participate, possibly because they were still actively involved in the pandemic response. We also acknowledge that PLWNCDs themselves were not included as participants in this study and recommend further research to learn from and respond to their experiences of the pandemic.

## Conclusions

The lessons around factors affecting continuity of care for NCDs and successful adaptations to care delivery in the context of COVID-19 are important for preparing for future health service disruptions, including in contexts experiencing ongoing crises or where marginalised or vulnerable communities have limited access to care. Our study findings reenforce global calls for more investment, strengthened partnerships and greater integration of NCDs into emergency preparedness, and building of resilient health systems.

## Supplementary Information

Supplementary Material 1.

Supplementary Material 2.

Supplementary Material 3.

Supplementary Material 4.

## Data Availability

The datasets used and/or analysed during the current study are available from the corresponding author on reasonable request.

## Abbreviations

CHW: Community Health Worker or Volunteer
CFIR: Consolidated Framework for Implementation Research
COVID-19: SARS CoV-2 Coronavirus
DM: Diabetes Mellitus
GACD: Global Alliance for Chronic Diseases
HTN: Hypertension
IPC: Infection Prevention and Control
LMIC: Low- and Middle-Income Country
LSHTM: London School of Hygiene & Tropical Medicine
NCDs: Non-communicable Diseases
NGO: Non-governmental Organisation
PLWNCDs: People Living with Non-communicable Disease
PPE: Personal Protective Equipment
UN: United Nations
UNHCR: United Nations High Commissioner for Refugees
WHO: World Health Organization

## Data availability

The data that support the findings of this study are not openly available due to reasons of sensitivity and are available from the corresponding author upon reasonable request. Data are located in controlled access data storage at the London School of Hygiene & Tropical Medicine.

## References

[1] Al-Oraibi A, Nellums LB, Chattopadhyay K. COVID-19, conflict, and non-communicable diseases among refugees. eClinicalMedicine. 2021;34. Available from: https://www.thelancet.com/journals/eclinm/article/PIIS2589-5370(21)00093-6/fulltext. Cited 14 Feb 2023. 10.1016/j.eclinm.2021.100813 PMC8008091 33817613

[2] Booth A, Reed AB, Ponzo S, Yassaee A, Aral M, Plans D, et al. Population risk factors for severe disease and mortality in COVID-19: A global systematic review and meta-analysis. PLoS ONE. 2021;16:e0247461. 33661992 10.1371/journal.pone.0247461 PMC7932512

[3] Kluge HHP, Wickramasinghe K, Rippin HL, Mendes R, Peters DH, Kontsevaya A, et al. Prevention and control of non-communicable diseases in the COVID-19 response. The Lancet. 2020;395:1678–80. 10.1016/S0140-6736(20)31067-9 PMC7211494 32401713

[4] Sheldon TA, Wright J. Twin epidemics of covid-19 and non-communicable disease. BMJ. 2020;369: m2618. 32605906 10.1136/bmj.m2618

[5] Horton R. Offline: COVID-19 is not a pandemic. The Lancet. 2020;396:874. 10.1016/S0140-6736(20)32000-6 PMC7515561 32979964

[6] World Health Organization. Non communicable diseases: Key Facts. 2022. Available from: https://www.who.int/news-room/fact-sheets/detail/noncommunicable-diseases. Cited 31 Oct 2022.

[7] Jawad M, Vamos EP, Najim M, Roberts B, Millett C. Impact of armed conflict on cardiovascular disease risk: a systematic review. Heart Br Card Soc. 2019;105:1388–94. 10.1136/heartjnl-2018-314459 31138670

[8] Ngaruiya C, Bernstein R, Leff R, Wallace L, Agrawal P, Selvam A, et al. Systematic review on chronic non-communicable disease in disaster settings. BMC Public Health. 2022;22:1234. 35729507 10.1186/s12889-022-13399-z PMC9210736

[9] UNHCR. UNHCR Global Trends 2023. UNHCR. 2023. Available from: https://www.unhcr.org/ie/global-trends. Cited 17 Feb 2024.

[10] OCHA. OCHA’s Strategic Plan 2023–2026: Transforming Humanitarian Coordination | OCHA. 2023. Available from: https://www.unocha.org/publications/report/world/ochas-strategic-plan-2023-2026-transforming-humanitarian-coordination. Cited 13 Dec 2023.

[11] UNHCR. Global Trends Report 2022. UNHCR. 2022 [cited 2023 Dec 13]. Available from: https://www.unhcr.org/global-trends-report-2022. Cited 13 Dec 2023.

[12] World Health Organisation. WHO | Global Action Plan for the Prevention and Control of NCDs 2013–2020. WHO. 2015. Available from: https://www.who.int/nmh/events/ncd_action_plan/en/. Cited 17 Feb 2015.

[13] Ansbro É, Issa R, Willis R, Blanchet K, Perel P, Roberts B. Chronic NCD care in crises: A qualitative study of global experts’ perspectives on models of care for hypertension and diabetes in humanitarian settings. J Migr Health. 2022;5:100094. 35434681 10.1016/j.jmh.2022.100094 PMC9010603

[14] Sachs JD, Karim SSA, Aknin L, Allen J, Brosbøl K, Colombo F, et al. The Lancet Commission on lessons for the future from the COVID-19 pandemic. The Lancet. 2022;400:1224–80. 10.1016/S0140-6736(22)01585-9 PMC9539542 36115368

[15] Luciani S, Caixeta R, Chavez C, Ondarsuhu D, Hennis A. What is the NCD service capacity and disruptions due to COVID-19? Results from the WHO non-communicable disease country capacity survey in the Americas region. BMJ Open. 2023;13: e070085. 36863746 10.1136/bmjopen-2022-070085 PMC9990165

[16] Yaacoub S, Zmeter C, Abbas LA, Leresche E, Kdouh O, Hammoud R, et al. Has the COVID-19 pandemic changed the utilization and provision of essential health care services from 2019 to 2020 in the primary health care network in Lebanon? Results from a nationwide representative cross-sectional survey. PLoS ONE. 2023;18: e0288387. 37440540 10.1371/journal.pone.0288387 PMC10343078

[17] World Health Organization. Pulse survey on continuity of essential health services during the COVID-19 pandemic: interim report, 27 August 2020. 2020. Available from: https://www.who.int/publications-detail-redirect/WHO-2019-nCoV-EHS_continuity-survey-2020.1. Cited 31 Oct 2022.

[18] World Health Organization. Non-communicable Diseases Progress Monitor 2022. 2022. Available from: https://www.who.int/publications-detail-redirect/9789240047761. Cited 12 Nov 2022.

[19] Nikoloski Z, Alqunaibet AM, Alfawaz RA, Almudarra SS, Herbst CH, El-Saharty S, et al. Covid-19 and non-communicable diseases: evidence from a systematic literature review. BMC Public Health. 2021;21:1068. 34090396 10.1186/s12889-021-11116-w PMC8178653

[20] The World Health Organization. The impact of the COVID-19 pandemic on noncommunicable disease resources and services: results of a rapid assessment. 2020 Sep. Available from: https://www.who.int/publications-detail-redirect/9789240010291

[21] Holland D, Heald AH, Stedman M, Hanna F, Wu P, Duff C, et al. Assessment of the effect of the COVID-19 pandemic on UK HbA1c testing: implications for diabetes management and diagnosis. J Clin Pathol. 2021; Available from: https://jcp.bmj.com/content/early/2021/10/12/jclinpath-2021-207776. Cited 15 Feb 2023. 10.1136/jclinpath-2021-207776 34645702

[22] Carr MJ, Wright AK, Leelarathna L, Thabit H, Milne N, Kanumilli N, et al. Impact of COVID-19 on diagnoses, monitoring, and mortality in people with type 2 diabetes in the UK. Lancet Diabetes Endocrinol. 2021;9:413. 33989537 10.1016/S2213-8587(21)00116-9 PMC8112824

[23] Del Pinto R, Ferri C, Mammarella L, Abballe S, Dell’Anna S, Cicogna S, et al. Increased cardiovascular death rates in a COVID-19 low prevalence area. J Clin Hypertens. 2020;22:1932–5. 10.1111/jch.14013 PMC7461222 32815667

[24] Wright FL, Cheema K, Goldacre R, Hall N, Herz N, Islam N, et al. Effects of the COVID-19 pandemic on secondary care for cardiovascular disease in the UK: an electronic health record analysis across three countries. Eur Heart J - Qual Care Clin Outcomes. 2023;9:377–88. 36385522 10.1093/ehjqcco/qcac077 PMC10284263

[25] Miller L, Alani AH, Avril N, Jingree ML, Atwiine AB, Amire KA, et al. Adaptation of care for non-communicable diseases during the COVID-19 pandemic: a global case study. BMJ Glob Health. 2022;7: e006620. 35798439 10.1136/bmjgh-2021-006620 PMC9263348

[26] World Health Organization. Strengthening health emergency prevention, preparedness, response and resilience. 2023. Available from: https://cdn.who.int/media/docs/default-source/emergency-preparedness/who_hepr_wha2023-21051248b.pdf?sfvrsn=a82abdf4_3&download=true. Cited 17 Feb 2024.

[27] Jaung MS, Willis R, Sharma P, Aebischer Perone S, Frederiksen S, Truppa C, et al. Models of care for patients with hypertension and diabetes in humanitarian crises: a systematic review. Health Policy Plan. 2021;36:509–32. 33693657 10.1093/heapol/czab007 PMC8128021

[28] Kessner DM, Kalk CE, Singer J. Assessing Health Quality — The Case for Tracers. N Engl J Med. 1973;288:189–94. 4682231 10.1056/NEJM197301252880406

[29] Lindström K, Berg L, Rylander B, Hagman A, Olsson L, Bengtsson C. A model for quality assessment in primary health care using the tracer condition technique with insulin treated diabetes as one of the tracers. Scand J Prim Health Care. 1997;15:92–6. 9232710 10.3109/02813439709018494

[30] Nolte E, Bain C, McKee M. Diabetes as a Tracer Condition in International Benchmarking of Health Systems. Diabetes Care. 2006;29:1007–11. 16644629 10.2337/diacare.2951007

[31] Damschroder LJ, Aron DC, Keith RE, Kirsh SR, Alexander JA, Lowery JC. Fostering implementation of health services research findings into practice: a consolidated framework for advancing implementation science. Implement Sci. 2009;4:50. 19664226 10.1186/1748-5908-4-50 PMC2736161

[32] Research Team-Center for Clinical Management Research. The Consolidated Framework for Implementation Research – Technical Assistance for users of the CFIR framework. 2022. Available from: https://cfirguide.org/. Cited 11 Nov 2022.

[33] StataCorp. Stata Statistical Software: Release 18. College Station, TX: StataCorp LLC.; 2023. https://www.stata.com/support/faqs/resources/citing-softwaredocumentation-faqs/.

[34] Gale NK, Heath G, Cameron E, Rashid S, Redwood S. Using the framework method for the analysis of qualitative data in multi-disciplinary health research. BMC Med Res Methodol. 2013;13:117. 24047204 10.1186/1471-2288-13-117 PMC3848812

[35] Cragg, L, Davies, M, Macdowall W. Health Promotion Theory. 2013. Available from: https://www.mheducation.co.uk/health-promotion-theory-9780335263202-emea-group. Cited 17 Feb 2024.

[36] Washington CH, Tyler FJ, Davis J, Shapiro DR, Richards A, Richard M, et al. Trauma training course: innovative teaching models and methods for training health workers in active conflict zones of Eastern Myanmar. Int J Emerg Med. 2014;7:46. 25624953 10.1186/s12245-014-0046-z PMC4298949

[37] Perri HB, Ed. National Community Health Worker Programs from Afghanistan to Zimbabwe. 2020. https://chwcentral.org/resources/health-for-the-people%E2%80%8B-national-community-health-worker-programs-from-afghanistan-to-zimbabwe. Cited 17 Feb 2024.

[38] Farzadfar F, Murray CJ, Gakidou E, Bossert T, Namdaritabar H, Alikhani S, et al. Effectiveness of diabetes and hypertension management by rural primary health-care workers (Behvarz workers) in Iran: a nationally representative observational study. The Lancet. 2012;379:47–54. 10.1016/S0140-6736(11)61349-4 22169105

[39] Newman PM, Franke MF, Arrieta J, Carrasco H, Elliott P, Flores H, et al. Community health workers improve disease control and medication adherence among patients with diabetes and/or hypertension in Chiapas, Mexico: an observational stepped-wedge study. BMJ Glob Health. 2018;3: e000566. 29527344 10.1136/bmjgh-2017-000566 PMC5841495

[40] Neupane B. Integrating nutrition in local governance structures: An example from suaahara program Nepal. FASEB J Conf Exp Biol. 2015;29.1. Supplement.741.3. 10.1096/FASEBJ.29.1_SUPPLEMENT.741.3.

[41] National Health Mission India. Module for Multi-Purpose Workers (MPW) - Female/Male on Prevention, Screening and Control of Common Non-Communicable Diseases. Available from: https://main.mohfw.gov.in/sites/default/files/Module%20for%20Multi-Purpose%20Workers%20-%20Prevention%2C%20Screening%20and%20Control%20of%20Common%20NCDS_2.pdf. Cited 17 Feb 2024.

[42] Salve S, Raven J, Das P, Srinivasan S, Khaled A, Hayee M, et al. Community health workers and Covid-19: Cross-country evidence on their roles, experiences, challenges and adaptive strategies. PLOS Glob Public Health. 2023;3: e0001447. 36962877 10.1371/journal.pgph.0001447 PMC10022071

[43] Bouabida K, Lebouché B, Pomey M-P. Telehealth and COVID-19 Pandemic: An Overview of the Telehealth Use, Advantages, Challenges, and Opportunities during COVID-19 Pandemic. Healthcare. 2022;10:2293. 36421617 10.3390/healthcare10112293 PMC9690761

[44] Abd-Alrazaq A, Hassan A, Abuelezz I, Ahmed A, Alzubaidi MS, Shah U, et al. Overview of Technologies Implemented During the First Wave of the COVID-19 Pandemic: Scoping Review. J Med Internet Res. 2021;23: e29136. 34406962 10.2196/29136 PMC8767979

[45] Favas C, Ansbro É, Eweka E, Agarwal G, Lazo Porras M, Tsiligianni I, et al. Factors Influencing the Implementation of Remote Delivery Strategies for Non-Communicable Disease Care in Low- and Middle-Income Countries: A Narrative Review. Public Health Rev. 2022;0. Available from: https://www.ssph-journal.org/articles/ 10.3389/phrs.2022.1604583/full. Cited 17 Aug 2022. 10.3389/phrs.2022.1604583 PMC9272771 35832336

[46] Slama S, Kim H-J, Roglic G, Boulle P, Hering H, Varghese C, et al. Care of non-communicable diseases in emergencies. The Lancet. 2017;389:326–30. 10.1016/S0140-6736(16)31404-0 27637675

[47] Remme M, Narasimhan M, Wilson D, Ali M, Vijayasingham L, Ghani F, et al. Self care interventions for sexual and reproductive health and rights: costs, benefits, and financing. BMJ. 2019;365: l1228. 30936210 10.1136/bmj.l1228 PMC6441864

[48] Pan X-F, Yang J, Wen Y, Li N, Chen S, Pan A. Non-Communicable Diseases During the COVID-19 Pandemic and Beyond. Eng Beijing China. 2021;7:899–902. 10.1016/j.eng.2021.02.013 PMC8056943 33898076

[49] World Health Organization Regional Office for South-East Asia. Integration of NCD care in emergency response and preparedness. 2018. Available from: https://www.who.int/publications-detail-redirect/9789290226352. Cited 17 Feb 2024.

[50] Guterres A, Spiegel P. The state of the world’s refugees: adapting health responses to urban environments. JAMA. 2012;308:673–4. 22893161 10.1001/2012.jama.10161

