# Supplementary material for: Lessons from the COVID-19 pandemic to strengthen NCD care and policy in humanitarian settings: a mixed methods study exploring humanitarian actors’ experiences
Source: BMC Health Serv Res. 2024 Sep 17;24:1081. doi: 10.1186/s12913-024-11458-2 (PMC11406764; doi:10.1186/s12913-024-11458-2)
Supplement: Supplementary file 1 — Supplementary Material 1. [file 12913_2024_11458_MOESM1_ESM.pdf]

SHARE: Management of non-communicable diseases in humanitarian settings during COVID-19 (copy)

Page 1: General information

Survey on non-communicable disease care during the current COVID-19 pandemic in humanitarian settings* in low- and middle-income countries

What is this survey about?

- The Covid-19 pandemic has caused huge disruption and prompted adaptation and innovation in health care delivery worldwide. We are interested in how services for chronic disease in already fragile humanitarian settings have coped with this pressure.
- We would like to understand how humanitarian organisations managed diabetes and/or hypertension (DM/HTN) before and during the pandemic.
- We are especially interested in what adaptations were made, what worked and what did not work.
- We aim to produce guidance on maintaining DM/HTN care when health care delivery is disrupted.

Who is doing the survey?

The Global Alliance for Chronic Disease (GACD) and London School of Hygiene and Tropical Medicine (LSHTM)

Who should fill it out?

- someone **at country or project level** with knowledge of **programmes/projects providing DM/HTN care in community, primary care or outpatient** contexts and how they have been impacted by Covid-19 (e.g. medical coordinator/health delegate/project coordinator/project medical responsable) or someone at headquarter level with detailed knowledge of the project/programme.
- Please forward the survey to relevant colleagues and contacts both inside and outside

of your organisation.

How long will it take? This survey should take about **20 minutes** to complete.

Further information and contacting you:

- We may wish to contact you to learn more about your responses.
- We will not share your contact details or individual responses without prior consent.
- For more information, please dowload the information sheet below and contact Éimhín Ansbro with questions: eimhin.ansbro@lshtm.ac.uk

Thank you for your help!

*We define humanitarian settings as: sites which are the focus of humanitarian action, such as acute, protracted or post-conflict zones; sites with disease outbreaks or affected by natural disasters; and areas hosting forcibly displaced populations (refugees and internally displaced persons displaced by famine, conflict or natural disaster)

Click here to download the information sheet

Page 2: Personal information

What is your name? *Optional*

What is your email address? *Optional*

Please enter a valid email address.

What is the full name of your organisation? ✱ *Required*

What is your job title? *Optional*

In which country are you currently working? ✱ *Required*

Page 3: Preamble

Does your organisation provide diabetes and/or hypertension (DM/HTN) care in LMIC humanitarian context(s)? This may involve direct care provision or support of other organisations/national health system. It may be community or health facility based. LMICs: low- and middle-income countries. ✱ *Required*

- Yes
- No

Page 4: Preamble

Does your organisation provide community-based and/or outpatient DM/HTN health care in any project sites **in the country where you work now?** This may involve direct provision or support of other organisations/national health system. ✱ *Required*

- ◯ Yes
- ◯ No

Page 5: Programme details

Please **answer the survey about one specific humanitarian project/programme** that includes care for diabetes and/or hypertension in the country where you work now. This includes projects where DM/HTN care is integrated with care for other conditions.

Programme/project name and location e.g. Irbid NCD project: *Optional*

What type of humanitarian context(s) and target population does this programme involve? ***Please select ALL that apply*** ✱ *Required*

Please select at least 1 answer(s).

- ☐ Natural disaster (e.g. earthquake, flood)
- ☐ Acute conflict
- ☐ Chronic or protracted conflict
- ☐ Public health emergency (e.g. measles, HIV, Ebola, malnutrition)
- ☐ Don't know/not sure
- ☐ Other origin to the crisis
- ☐ Refugee setting
- ☐ Internally displaced setting
- ☐ Host population
- ☐ Returnees
- ☐ Other population

If you selected Other, please specify:

In what type of location does this programme/project operate and how does it interact with the host country public health system? *Please select ALL that apply* ✱ *Required*

Please select at least 1 answer(s).

- [ ] Urban / peri-urban
- [ ] Rural
- [ ] Camp settlement
- [ ] NON-camp settlement
- [ ] Other type of location
- [ ] Stand-alone, humanitarian organisation-run (not integrated)
- [ ] Humanitarian organisation-run (substitution) within a public health facility
- [ ] Humanitarian organisation support of public health services within public health facility
- [ ] Referral to other public health services
- [ ] Referral to private services
- [ ] Other referral pathways

If you selected Other, please specify:

Is this programme: *select ALL that apply* ✱ *Required*

- ○ Providing/supporting care for diabetes only
- ○ Providing/supporting care for hypertension only

- ○ Providing/supporting care for diabetes and hypertension (only)
- ○ Integrated with care for other NCDs e.g. cardiovascular disease, mental health care
- ○ Integrated with other specific services e.g. HIV/TB/MCH
- ○ Integrated within general primary health care (PHC) services
- ○ Don’t know/not sure
- ● Other

If you selected Other, please specify:

Please specify which other NCDs:

Please specify which other services:

Which type of Diabetes is included: *select ALL that apply*

- ○ Type 1 only
- ○ Type 2 only
- ○ Types 1 and 2
- ○ Gestational diabetes
- ○ Don't know/not sure

Page 6: Diabetes/Hypertension management: medical consultation

In this section, we would like to understand what, how, where and by whom **medical consultation** for DM/HTN was delivered ***BEFORE and DURING the pandemic***

Did your programme/project provide DM/HTN medical consultation BEFORE and/or DURING the pandemic? Please select one option only

- ○ Yes, BEFORE the pandemic and partially or fully maintained DURING the pandemic
- ○ Yes, BEFORE the pandemic but completely stopped DURING the pandemic
- ○ No, not BEFORE nor DURING the pandemic
- ○ No, not BEFORE but introduced since the pandemic

Did/does medical consultation involve prescription and/or dispensing of medications?

- ☐ Yes
- ☐ No

Please specify what is involved in medication prescription and/or dispensing? Select ALL that apply

- ☐ Initiation of medications
- ☐ Adjustment of medications
- ☐ No medication adjustment (e.g. consultation by nurses/CHW with no prescribing rights)
- ☐ Dispensing of medications
- ☐ Provision of free medications
- ☐ Co-payment for medications
- ☐ Oral medications

- [ ] Insulin
- [ ] Insulin syringes and needles
- [ ] Insulin pen devices

How was the provision of medical consultation affected since the pandemic? Please select one option only

- ○ Continued unchanged (in terms of services provided and modalities of delivery)
- ○ Adapted
- ○ Suspended temporarily (and unchanged when provided)
- ○ Suspended temporarily (and adapted when provided)
- ○ Completely stopped

Who was providing medical consultation BEFORE the Pandemic? Please select ALL that apply (e.g. stable patient are seen by a lay health worker whereas patients with complications are seen by a nurse or doctor).

- [ ] Generalist doctors
- [ ] Specialist doctors
- [ ] Nurses
- [ ] Lay health worker (facility based)
- [ ] Community health workers
- [ ] Community volunteers
- [ ] Other

If you selected Other, please specify:

Who was/is providing medical consultation DURING the pandemic? If it is unchanged from before the pandemic, select "unchanged". If medical consultation has been adapted or newly introduced, select all that apply.

- Unchanged
- Generalist doctors
- Specialist doctors
- Nurses
- Lay health worker (facility based)
- Community health workers
- Community volunteers
- Other

If you selected Other, please specify:

How was medical consultation provided BEFORE the pandemic? Please select ALL that apply

- [ ] Face-to-face individual services
- [ ] Face-to-face group services
- [ ] Telephone consultations
- [ ] Video consultations
- [ ] Mobile phone patient services (e.g. text/SMS/Application)
- [ ] Technology supporting health care workers (e.g. decision support/telemedicine)
- [ ] Peer support/community adherence groups
- [ ] Other

If you selected Other, please specify:

How was/is medical consultation delivered DURING the pandemic? If it is unchanged from before the pandemic, select "unchanged". If medical consultation delivery has been adapted or

newly introduced, select the appropriate option(s).

- [ ] Unchanged
- [ ] Face-to-face individual services
- [ ] Face-to-face group services
- [ ] Peer support/community adherence groups
- [ ] Telephone consultations
- [ ] Video consultations
- [ ] Mobile phone patient services (e.g. text/SMS/Application)
- [ ] Technology supporting health care workers (e.g. decision support/telemedicine)
- [ ] Other

If you selected Other, please specify:

____

Where was medical consultation delivered BEFORE the pandemic? Please select ALL that apply

- [ ] Mobile medical unit / mobile clinics
- [ ] Primary health care posts or clinics
- [ ] Secondary or tertiary clinics or hospitals
- [ ] Community (e.g. in a community hub)
- [ ] Home visit (e.g. home visit team/community health volunteer)
- [ ] Other

If you selected Other, please specify:

____

Where was/is medical consultation delivered DURING the pandemic? If it is unchanged from before the pandemic, select "unchanged". If medical consultation delivery location has been

adapted or newly introduced, select the appropriate option(s).

- ☐ Unchanged
- ☐ Mobile medical unit / mobile clinics
- ☐ Primary health care posts or clinics
- ☐ Secondary or tertiary clinics or hospitals
- ☐ Community (e.g. in a community hub)
- ☐ People’s homes (e.g. home visit team/community health volunteer)
- ☐ All services virtual (not face-to-face)
- ☐ Other

If you selected Other, please specify:

Page 7: Diabetes/Hypertension management: disease monitoring

In this section, we would like to understand what, how, where and by whom **disease monitoring** for DM/HTN was delivered ***BEFORE and DURING the pandemic***

Did your programme/project provide DM/HTN disease monitoring BEFORE and DURING the pandemic? Please select one option only

- ◯ Yes, BEFORE the pandemic and partially or fully maintained DURING the pandemic
- ◯ Yes, BEFORE the pandemic but completely stopped DURING the pandemic
- ◯ No, not BEFORE nor DURING the pandemic
- ◯ No, not BEFORE but introduced since the pandemic

Please specify what was/is involved in disease monitoring services provided by your organisation. Select ALL that apply

- ☐ Blood pressure monitoring
- ☐ Blood glucose check with glucometer
- ☐ Continuous glucose monitoring e.g. FreeStyle Libre
- ☐ HbA1c monitoring
- ☐ Creatinine monitoring
- ☐ Cholesterol monitoring
- ☐ Proteinuria on urine dipstick
- ☐ Albumin creatinine ratio monitoring
- ☐ Diabetic retinopathy screening
- ☐ Diabetic foot check
- ☐ ECG
- ☐ Point-of-care tests
- ☐ External laboratory tests

- [ ] Other
- [ ] Other

If you selected Other, please specify:

How was the provision of disease monitoring services affected since the pandemic?
Please select one option only

- ○ Continued unchanged (in terms of services provided and modalities of delivery)
- ○ Adapted
- ○ Suspended temporarily (and unchanged when resumed)ed)
- ○ Suspended temporarily (and adapted when resumed)
- ○ Completely stopped

Who was providing disease monitoring services BEFORE the Pandemic? Please select ALL that apply

- [ ] Generalist doctors
- [ ] Specialist doctors
- [ ] Nurses
- [ ] Lay health workers (facility based)
- [ ] Community health workers
- [ ] Community volunteers
- [ ] Peer supporters
- [ ] Counsellor
- [ ] Other

If you selected Other, please specify:

Who was/is providing disease monitoring services DURING the pandemic? If it is unchanged from before the pandemic, select "unchanged". If this has been adapted or newly introduced, select ALL that apply.

- ☐ Unchanged
- ☐ Generalist doctors
- ☐ Specialist doctors
- ☐ Nurses
- ☐ Lay health workers (facility based)
- ☐ Community health workers
- ☐ Community volunteers
- ☐ Peer supporters
- ☐ Counsellor
- ☐ Other

If you selected Other, please specify:

How were disease monitoring services provided BEFORE the pandemic? Please select ALL that apply

- ☐ Face-to-face individual services
- ☐ Face-to-face group services
- ☐ Telephone consultations
- ☐ Video consultations
- ☐ Mobile phone patient services (e.g. text/SMS/Application)
- ☐ Technology supporting health care workers (e.g. decision support/telemedicine)
- ☐ Peer support/community adherence groups

- [ ] Other

If you selected Other, please specify:

How were/are disease monitoring services delivered DURING the pandemic? If it is unchanged from before the pandemic, select "unchanged". If services delivery has been adapted or newly introduced, select the appropriate option(s).

- [ ] Unchanged
- [ ] Face-to-face individual services
- [ ] Face-to-face group services
- [ ] Peer support/community adherence groups
- [ ] Telephone consultations
- [ ] Video consultations
- [ ] Mobile phone patient services (e.g. text/SMS/Application)
- [ ] Technology supporting health care workers (e.g. decision support/telemedicine)
- [ ] Other

If you selected Other, please specify:

Where were disease monitoring services delivered BEFORE the pandemic? Please select ALL that apply

- [ ] Mobile medical unit / mobile clinics
- [ ] Primary health care posts or clinics
- [ ] Secondary or tertiary clinics or hospitals
- [ ] Community (e.g. in a community hub)
- [ ] People’s homes (e.g. home visit team/community health volunteer)

- [ ] External laboratory
- [ ] Other

If you selected Other, please specify:

Where were/are disease monitoring services delivered DURING the pandemic? If it is unchanged from before the pandemic, select "unchanged". If services delivery location has been adapted or newly introduced, select the appropriate option(s).

- [ ] Unchanged
- [ ] Mobile medical unit / mobile clinics
- [ ] Primary health care posts or clinics
- [ ] Secondary or tertiary clinics or hospitals
- [ ] Community (e.g. in a community hub)
- [ ] People’s homes (e.g. home visit team/community health volunteer)
- [ ] Other

If you selected Other, please specify:

Page 8: Diabetes/Hypertension management: patient education & support

In this section, we would like to understand what, how, where and by whom **patient education & support services** for DM/HTN were delivered ***BEFORE and DURING the pandemic***

Did your programme/project provide DM/HTN patient education & support services BEFORE the pandemic? Please select one option only

- ○ Yes, BEFORE the pandemic and partially or fully maintained DURING the pandemic
- ○ Yes, BEFORE the pandemic but completely stopped DURING the pandemic
- ○ No, not BEFORE nor DURING the pandemic
- ○ No, not BEFORE but introduced since the pandemic

Please specify what was/is involved in patient education & support services provided by your organisation. Select ALL that apply

- ☐ Patient education and support (health living, disease literacy, medication adherence)
- ☐ Patient self-management skills training (e.g. self-monitoring of blood pressure/glucose)
- ☐ Mental health and psychosocial support
- ☐ Other
- ☐ Other

If you selected Other, please specify:

How was the provision of patient education & support services affected since the pandemic? Please select one option only

- ○ Continued unchanged (in terms of services provided and modalities of delivery)
- ○ Adapted
- ○ Suspended temporarily (and unchanged when resumed)
- ○ Suspended temporarily (and adapted when resumed)
- ○ Completely stopped

Who was providing patient education & support services BEFORE the Pandemic? Please select ALL that apply

- ☐ Generalist doctors
- ☐ Specialist doctors
- ☐ Nurses
- ☐ Lay health workers (facility based)
- ☐ Community health workers
- ☐ Community volunteers
- ☐ Peer supporters
- ☐ Counsellor
- ☐ Other

If you selected Other, please specify:

Who was/is providing patient education & support services DURING the pandemic? If it is unchanged from before the pandemic, select "unchanged". If this has been adapted or newly introduced, select ALL that apply.

- ☐ Unchanged

- [ ] Generalist doctors
- [ ] Specialist doctors
- [ ] Nurses
- [ ] Lay health workers (facility based)
- [ ] Community health workers
- [ ] Community volunteers
- [ ] Peer supporters
- [ ] Counsellor
- [ ] Other

If you selected Other, please specify:

____

How were patient education & support services provided BEFORE the pandemic? Please select ALL that apply

- [ ] Face-to-face individual services
- [ ] Face-to-face group services
- [ ] Telephone consultations
- [ ] Video consultations
- [ ] Mobile phone patient services (e.g. text/SMS/Application)
- [ ] Technology supporting health care workers (e.g. decision support/telemedicine)
- [ ] Peer support/community adherence groups
- [ ] Other

If you selected Other, please specify:

____

How were/are patient education & support services delivered DURING the pandemic? If

it is unchanged from before the pandemic, select "unchanged". If services delivery has been adapted or newly introduced, select the appropriate option(s).

- ☐ Unchanged
- ☐ Face-to-face individual services
- ☐ Face-to-face group services
- ☐ Peer support/community adherence groups
- ☐ Telephone consultations
- ☐ Video consultations
- ☐ Mobile phone patient services (e.g. text/SMS/Application)
- ☐ Technology supporting health care workers (e.g. decision support/telemedicine)
- ☐ Other

If you selected Other, please specify:

____

Where were patient education & support services delivered BEFORE the pandemic?
Please select ALL that apply

- ☐ Mobile medical unit / mobile clinics
- ☐ Primary health care posts or clinics
- ☐ Secondary or tertiary clinics or hospitals
- ☐ Community (e.g. in a community hub)
- ☐ People’s homes (e.g. home visit team/community health volunteer)
- ☐ Other

If you selected Other, please specify:

____

Where were/are patient education & support services delivered DURING the

pandemic? If it is unchanged from before the pandemic, select "unchanged". If services delivery location has been adapted or newly introduced, select the appropriate option(s).

- ☐ Unchanged
- ☐ Mobile medical unit / mobile clinics
- ☐ Primary health care posts or clinics
- ☐ Secondary or tertiary clinics or hospitals
- ☐ Community (e.g. in a community hub)
- ☐ People’s homes (e.g. home visit team/community health volunteer)
- ☐ Other

If you selected Other, please specify:

Page 9: Diabetes/Hypertension management: primary prevention & community screening

In this section, we would like to understand what, how, where and by whom **primary prevention & community screening services*** for DM/HTN were delivered ***BEFORE and DURING the pandemic***

*Primary prevention refers to prevention activities targeting population groups without the disease. Community screening refers to screening of those previously undiagnosed in the community.

Did your programme/project provide DM/HTN primary prevention & community screening services BEFORE the pandemic? Please select one option only

- ◯ Yes, BEFORE the pandemic and partially or fully maintained DURING the pandemic
- ◯ Yes, BEFORE the pandemic but completely stopped DURING the pandemic
- ◯ No, not BEFORE nor DURING the pandemic
- ◯ No, not BEFORE but introduced since the pandemic

Please specify what was/is involved in primary prevention & community screening services provided by your organisation. Select ALL that apply

- ☐ Community education & sensitisation activities
- ☐ Community based prevention activities e.g sport, cooking classes
- ☐ Community screening for DM/HTN (in those previously undiagnosed)

How was the provision of primary prevention & community screening services affected since the pandemic? Please select one option only

- ◯ Continued unchanged (in terms of services provided and modalities of delivery)
- ◯ Adapted
- ◯ Suspended temporarily (and unchanged when provided)

- Suspended temporarily (and adapted when provided)
- Completely stopped

Who was providing primary prevention & community screening services BEFORE the Pandemic? Please select ALL that apply

- [ ] Generalist doctors
- [ ] Specialist doctors
- [ ] Nurses
- [ ] Lay health workers (facility based)
- [ ] Community health workers
- [ ] Community volunteers
- [ ] Peer supporters
- [ ] Counsellor
- [ ] Other

If you selected Other, please specify:

Who was/is providing primary prevention & community screening services DURING the pandemic? If it is unchanged from before the pandemic, select "unchanged". If this has been adapted or newly introduced, select ALL that apply.

- [ ] Unchanged
- [ ] Generalist doctors
- [ ] Specialist doctors
- [ ] Nurses
- [ ] Lay health workers (facility based)
- [ ] Community health workers
- [ ] Community volunteers
- [ ] Peer supporters

- [ ] Counsellor
- [ ] Other

If you selected Other, please specify:

[ ]

How were primary prevention & community screening services provided BEFORE the pandemic? Please select ALL that apply

- [ ] Face-to-face individual services
- [ ] Face-to-face group services
- [ ] Telephone consultations
- [ ] Video consultations
- [ ] Mobile phone patient services (e.g. text/SMS/Application)
- [ ] Technology supporting health care workers (e.g. decision support/telemedicine)
- [ ] Peer support/community adherence groups
- [ ] Other

If you selected Other, please specify:

[ ]

How were/are primary prevention & community screening services delivered DURING the pandemic? If it is unchanged from before the pandemic, select "unchanged". If services delivery has been adapted or newly introduced, select ALL that apply.

- [ ] Unchanged
- [ ] Face-to-face individual services
- [ ] Face-to-face group services
- [ ] Peer support/community adherence groups
- [ ] Telephone consultations

- [ ] Video consultations
- [ ] Mobile phone patient services (e.g. text/SMS/Application)
- [ ] Technology supporting health care workers (e.g. decision support/telemedicine)
- [ ] Other

If you selected Other, please specify:

Where were primary prevention & community screening services delivered BEFORE the pandemic? Please select ALL that apply

- [ ] Mobile medical unit / mobile clinics
- [ ] Primary health care posts or clinics
- [ ] Secondary or tertiary clinics or hospitals
- [ ] Community (e.g. in a community hub)
- [ ] People's homes (e.g. home visit team/community health volunteer)
- [ ] Other

If you selected Other, please specify:

Where were/are primary prevention & community screening services delivered DURING the pandemic? If it is unchanged from before the pandemic, select "unchanged". If services delivery location has been adapted or newly introduced, select the ALL that apply.

- [ ] Unchanged
- [ ] Mobile medical unit / mobile clinics
- [ ] Primary health care posts or clinics
- [ ] Secondary or tertiary clinics or hospitals
- [ ] Community (e.g. in a community hub)

- [ ] People’s homes (e.g. home visit team/community health volunteer)
- [ ] Other

If you selected Other, please specify:

Page 10: Changes to DM/HTN services and context DURING the COVID-19 pandemic

During the pandemic, if you had to **stop or suspend any of the DM/HTN services** that were provided before the pandemic, why was this necessary? *Please select ALL that apply*
✱ *Required*

- ☐ We did not suspend DM/HTN services due to the pandemic
- ☐ Insufficient lay staff to support routine care
- ☐ Insufficient medical staff to support routine care
- ☐ Interrupted medication supply/ stock outs
- ☐ Insufficient personal protective equipment (PPE)
- ☐ Disruption to physicial access to services
- ☐ Movement restricted due to government guidelines
- ☐ Poor internet coverage
- ☐ Poor phone coverage
- ☐ Patient fear of face-to-face attendance
- ☐ Stopped temporarily while adaptations made
- ☐ Don’t know
- ☐ Other

If you selected Other, please specify:

Which (if any) of the following modifications/adaptations allowed you continue to deliver care for DM/HTN, or to facilitate patient self-management, **DURING** the pandemic?
Please select ALL that apply ✱ *Required*

- ☐ Reduced frequency of face-to-face medical consultation
- ☐ Adaptations to medications procurement/supply

- ☐ Adaptations to medication pick up/ delivery to patient
- ☐ Task sharing of certain tasks to facility-based staff
- ☐ Task sharing of certain tasks to community-based staff
- ☐ Telephone consultations
- ☐ Proactive phone calls using patient register
- ☐ Use of SMS text messages
- ☐ Use of smartphone apps for communication (e.g. Whatsapp)
- ☐ Use of other social media (Facebook, Instagram)
- ☐ Use of specific DM/HTN apps for patient self management
- ☐ Simplification of treatment algorithms
- ☐ Simplification/reduced frequency of lab testing/monitoring
- ☐ Telemedicine (remote clinical support for health workers)
- ☐ Decision support tools for health workers
- ☐ Don’t know
- ☐ Other

If you selected Other, please specify:

Could you provide more details about any adaptation/modifications made to continue providing DM/HTN services **DURING** the pandemic? *If you did not make any modifications, please write “Not applicable”.* ✱ *Required*

What (if any) tools or guidance, did the project develop/use to facilitate adaptations in

response to the pandemic? *Optional*

What (if any) training of staff was provided in response to the pandemic? *(please briefly explain the topic, training method, duration, and cadre of health staff targeted)*

From you and your organisation's experience, what are some of the challenges patients face in managing their **DM/HTN** that were due to or exacerbated by the pandemic? *Please select ALL that apply* ✱ *Required*

- ☐ Physical restriction/ restricted movement
- ☐ Social restriction
- ☐ Financial hardship
- ☐ Limited healthy food options
- ☐ Limited access to medical consultation
- ☐ Limited access to medicines
- ☐ Inability to access disease monitoring
- ☐ Poor mental health (e.g., depression)
- ☐ Don't know
- ☐ Other
- ☐ Distrust of information related to pandemic
- ☐ Distrust of health services
- ☐ Fear of attending healthcare services

If you selected Other, please specify:

If your organisation **continues** to offer all or some DM/HTN services **DURING** the pandemic, what are some of the **internal / programmatic challenges** from the provider perspective? *Please select ALL that apply* ✱ *Required*

- ☐ Medication procurement/supply issues
- ☐ Staff workload/burnout
- ☐ Staff absence due COVID diagnosis or related quarantine/isolation
- ☐ Limited patient physical access/movement restrictions
- ☐ Patient fear restricting attendance at facility based appointments
- ☐ Unequal access to care; certain groups (e.g., women or elderly) are struggling to access care more than others
- ☐ Inability to monitor glucose remotely
- ☐ Inability to monitor blood pressure remotely
- ☐ Uncertainty making planning difficulty
- ☐ Violence against staff
- ☐ Don’t know
- ☐ Other
- ☐ Health resources diverted to Covid pandemic

If you selected Other, please specify:

If your organisation **continues** to offer DM/HTN services **DURING** the pandemic, what are some of the **external/contextual challenges**? *Please select ALL that apply* ✱ *Required*

- ☐ Financial implications of crisis
- ☐ Poor mobile phone coverage (non smartphone)
- ☐ Poor Smartphone availability among patients and health providers
- ☐ Internet connectivity issues
- ☐ Resistance among patients to adapt to telephone or video consultations
- ☐ Don't know
- ☐ Other

If you selected Other, please specify:

Please describe **any broader contextual changes in policy or practice** in reponse to the pandemic that affected (either positively or negatively) how the programme operated? *(e.g. changes to government policy, procurement, changes in communication or cooperation with other actors ?)*

Please explain any lessons learned in your efforts to respond to the challenges **DURING** the pandemic - what do you feel worked?

What do you feel didn't work and why?

Page 11: Additional information

LSHTM is launching a website to collate existing research, guidelines and practical tools to support the implementation of NCD care in humanitarian settings. Please select all that apply below.

- ☐ I would use an LSHTM NCDs in humanitarian settings website
- ☐ I would NOT use an LSHTM NCDs in humanitarian settings website
- ☐ An LSHTM NCDs in humanitarian settings website would be a useful addition to existing resources
- ☐ An LSHTM NCDs in humanitarian settings website would NOT be a useful addition to existing resources
- ☐ I would like to see up to date relevant research/evidence on the site
- ☐ I would like to see research tools e.g. qualitative topic guides on the site
- ☐ I would like to see clinical guidelines on the site
- ☐ I would like to see operational guidelines on the site
- ☐ I would like to see practical tools e.g. patient files on the site
- ☐ I already use OTHER websites/ resources (please select "other" and specify below)
- ☐ I would like to see OTHER uses for the site (please "other" and specify below)
- ☐ Other

If you selected Other, please specify:

I would like to receive further information about this project, including any resources, virtual events, or other outputs that may result (using the email address given above). ✱ *Required*

- ◯ Yes
- ◯ No

I agree to be contacted at the email address given above with follow-up questions about my survey answers. ✱ *Required*

- ◯ Yes
- ◯ No

Page 12: Final page

THANK YOU so much for taking the time to complete our survey! We sincerely appreciate your time, dedication, and participation in our online survey.

We will get in touch to share the findings of this survey!
