# Supplementary material for: Lessons from the COVID-19 pandemic to strengthen NCD care and policy in humanitarian settings: a mixed methods study exploring humanitarian actors’ experiences
Source: BMC Health Serv Res. 2024 Sep 17;24:1081. doi: 10.1186/s12913-024-11458-2 (PMC11406764; doi:10.1186/s12913-024-11458-2)
Supplement: Supplementary file 2 — Supplementary Material 2. [file 12913_2024_11458_MOESM2_ESM.docx]

**Annex 2: Topic guide: Follow-up interviews (post online survey)**

**Project title: Maintaining access to care for people with hypertension and/ or diabetes in humanitarian settings during COVID19 disruption**

**NB ACCESS to CARE –** could involve re-strategizing, reducing frequency or necessity for face-to-face attendance at a primary care facility through e.g.

- Technology supported remote care e.g. via telephone/mobile phone application/ SMS messaging
- Introduction / enhancement of community-based care by HCW
- Introduction / enhancement of peer support groups
- Strengthening self-care
- Minimising or adapting facility-based attendance e.g. increasing number of months of drugs dispensed, having family attend for drugs pick up, eliminating/adapting blood testing

**Tips for interviewers:**

- Start with general, open, questions - those in bold in the topic guide
- Give space to the interviewee to answer in their own words
- Use general prompting questions, such as “could you tell me a bit more about....; you mentioned x,y,z, earlier, could you expand on that...: could you explain to me a bit more about that...etc.”
- Use the specific prompts included in the topic guide, as needed, to gain more detailed responses
- Avoid asking “why” if possible as this can be seen as intimidating
- You do not need to follow the questions in the order they are presented in the topic guide or to cover all questions with each interviewee. If possible, try to prioritise those highlighted in blue.
- Before the interview, refer to the participant’s responses to the online survey.

**Welcome and introduction**

Prior to starting the interview:

1. Introduce yourself and reiterate the purpose of the study and the aim of this interview;
2. Give a brief outline of the interview and explain the interview process. Check with the participant their available time for the interview;
3. Ask if the participant has any questions or needs any clarifications;
4. Review the completed consent form with the participant;
5. Thank the participant for taking part.

**Participant information (warm-up questions)**

**Ask the participant to briefly introduce themselves (they can describe their professional background and current position in their organisation, and their main responsibilities).**

| **Topic** | **Questions / Prompts** |
| --- | --- |
| Effects of the COVID-19 pandemic on DM/HTN primary care | *Based on the participant’s responses on the DM/HTN services that their organisation was providing before the pandemic*   1. **Could you tell me about how the Covid-19 pandemic has affected you where you are working?**    1. How was the country affected at the beginning of the pandemic / at the time of the survey?    2. Did this change as the pandemic progressed? What about now? 2. **Could you describe how the Covid-19 pandemic affected how your project/programme provided services for DM/HTN?**    1. How was **access** to DM/HTN services affected? (for the target population / health staff / organisation staff)    2. How was patients’ **use of the services** affected by the pandemic? 3. **During the pandemic response, how were NCDs prioritised compared to other health or non-health services your organisation provides?**    1. Were NCDs de-prioritised compared to other activities e.g. the healthcare response to Covid?    2. Were NCD patients given greater priority because of their greater risk of morbidity/mortality from Covid 19? (and /or were NCD patients triaged in terms of severity of risk?) 4. **Thinking specifically about services for DM/HTN, did your project/programme reorganise or adapt the way these services were delivered in response to the pandemic?**   **Could you describe in more detail what changed?**   - 1. Which aspect of service delivery was adapted? *(Prompt who, what, where, how)* ?   2. Which specific issues were these adaptations expected to address (e.g. restrictions on travel/shielding NCD patients at home/lack of HCW)?   3. Which activities were prioritised or maintained?   4. Did you project/programme reduce or stop providing specific services/ activities for patients with DM/HTN because of the pandemic? If yes, which ones?  1. **How was the decision made about how to adapt the programme?**    1. Who made it?    2. What resources or evidence were used to make the decision? 2. **Were the needs and preferences of the NCD patients being served by your project/programme taken into account? If so, how?**    1. Were specific groups (if any) targeted by the programme’s adaptation? If yes, which group(s)?    2. Were any groups less likely to be able to access the intervention? (e.g. vulnerable groups, women, poor mobility, groups with poorer access to internet/phone, lower education attainment)    3. How was the identification of new cases of NCDs affected by the pandemic? Was this deprioritised or was this prioritised? |
| Adaptations made to maintain primary care for DM/HTN: Fit or coherence with existing services | 1. **How did the changes fit in with existing services or ways of working?** 2. **What kind of training, if any, did you and colleagues have before implementing these changes?**    1. Did you feel you had enough training; did you feel prepared? 3. **What resources did you and your colleagues have to support you in making these changes?**     1. What about training material, staffing, time, infrastructure and supplies?    2. Would anything else have helped you to implement the changes**?** |
| Adaptations made to maintain primary care for DM/HTN: challenges and facilitators to implementation, internal context | 1. **Could you tell us more about what worked well when you made these changes?**     1. What worked well for patients?    2. What worked well for providers?    3. What worked well for the organisation?    4. What factors helped to facilitate this success? (e.g. resources, expertise, experience)    5. Were there any unexpected benefits/outcomes? 2. **Could you tell me about any aspects of the changes that didn’t work so well?**     1. Did any aspects not work well for patients?    2. Did any aspects not work so well for providers?    3. What didn’t work well for the organisation itself?    4. What do you think were the main reasons why it didn’t work? What were the main barriers?    5. How has the project tried to address these challenges? |
| Adaptations made to maintain primary care for DM/HTN:external context | 1. **To what extent did you or your organisation work with other organisations (INGOs, local NGOs/CSOs, ethnic providers) to implement these changes?**    1. What organisations were involved? In what way did you work together?    2. How successful or otherwise do you think this collaboration was? Do you think it will/should continue? 2. **Could you tell me about how the broader local or national context influenced the adaptations made to your DMHTN services?**    1. What kind of local, state, or national Covid-pandemic related policies, regulations, or guidelines influenced the adaptations made? What about movement restrictions/lockdowns etc.    2. Could you tell me about any factors related to the context that facilitated the changes we have discussed? (e.g., local authorities buy-in & engagement, phone network, perception of target population etc.)?    3. Could you tell me about barriers related to the context (e.g., local authorities buy-in & engagement, phone network, perception of target population etc.)? |
|  | 1. **What is currently happening with those changes/adaptations?**    1. Did things return to how they were before the pandemic? If yes, when did this happen?    2. Are they still in place? Have any further adaptations been made [try to establish a timeline] 2. **What aspects do you think should be maintained or introduced into other NCD projects/programmes?** 3. **What are the key lessons you learned that you would like to share with others about your experience of providing NCD care during the Covid-19 pandemic?** |
| Close | *Do you have any questions for me at this point?*  *Thank you very much for your time.*  *You can contact me or the study lead if you have any further questions (eimhin.ansbro@lshtm.ac.uk)* |
