# Supplementary material for: Lessons from the COVID-19 pandemic to strengthen NCD care and policy in humanitarian settings: a mixed methods study exploring humanitarian actors’ experiences
Source: BMC Health Serv Res. 2024 Sep 17;24:1081. doi: 10.1186/s12913-024-11458-2 (PMC11406764; doi:10.1186/s12913-024-11458-2)
Supplement: Supplementary file 3 — Supplementary Material 3. [file 12913_2024_11458_MOESM3_ESM.docx]

| **Annex 3: How delivery of NCD service components changed during the COVID-19 pandemic** | | | | | | | | |
| --- | --- | --- | --- | --- | --- | --- | --- | --- |
|  | **Service Component** | | | | | | | |
|  | **Medical consultation** | | **Disease monitoring** | | **PLWNCDs education and support services** | | **Primary prevention and community screening** | |
| **Before or Change During the COVID-19 Pandemic->** | Before (%) | Change During (%) | Before (%) | Change During (%) | Before (%) | Change During (%) | Before (%) | Change During (%) |
| **Service provider** |  |  |  |  |  |  |  |  |
| Generalist doctors | 89 | 14 | 81 | 18 | 67 | 24 | 34 | 8 |
| Specialist doctors | 27 | 1 | 20 | 5 | 11 | 4 | 4 | 1 |
| Nurse | 41 | 1 | 54 | 13 | 64 | 28 | 48 | 9 |
| Lay health workers | 12 | 0 | 10 | 1 | 19 | 4 | 6 | 2 |
| Community Health Workers | 14 | 0 | 22 | 5 | 47 | 12 | 48 | 13 |
| Community volunteers | 7 | 0 | 10 | 4 | 20 | 9 | 19 | 8 |
| Other | 13 | 0 | 13 | 1 | 13 | 6 | 3 | 4 |
| Unchanged – same cadre before and during | N/A | 84 | N/A | 72 | N/A | 58 | N/A | 41 |
|  |  |  |  |  |  |  |  |  |
| **How the service was provided** |  |  |  |  |  |  |  |  |
| Face to face individual services | 93 | 40 | 99 | 34 | 95 | 48 | 91 | 39 |
| Face to face group services | 16 | 1 | 21 | 5 | 65 | 6 | 69 | 10 |
| Telephone consultations | 2 | 24 | 3 | 19 | 6 | 21 | 2 | 15 |
| Video consultations | 0 | 1 | 0 | 1 | 1 | 4 | 0 | 3 |
| Mobile phone PLWNCDs services | 1 | 9 | 3 | 9 | 3 | 16 | 2 | 13 |
| Technology supporting health care workers | 3 | 5 | 2 | 5 | 2 | 6 | 2 | 5 |
| Peer support/community adherence groups | 5 | 3 | 2 | 3 | 8 | 6 | 6 | 2 |
| Other | 2 | 9 | 4 | 8 | 7 | 7 | 8 | 8 |
|  |  |  |  |  |  |  |  |  |
| **Where the services were provided** |  |  |  |  |  |  |  |  |
| Mobile medical unit/mobile clinics | 15 | 3 | 12 | 2 | 15 | 6 | 12 | 4 |
| Primary health care posts or clinics | 89 | 34 | 86 | 33 | 87 | 39 | 58 | 25 |
| Secondary or tertiary clinics or hospitals | 32 | 11 | 35 | 8 | 31 | 9 | 14 | 4 |
| Community | 9 | 1 | 10 | 2 | 29 | 10 | 30 | 8 |
| Home visits | 25 | 12 | 25 | 12 | 39 | 15 | 35 | 10 |
| External laboratory | 0 | 0 | 8 | 0 | 0 | 0 | 0 | 0 |
| All services virtual | 0 | 4 | 0 | 0 | 0 | 0 | 0 | 0 |
| Other | 0 | 3 | 1 | 2 | 0 | 9 | 1 | 3 |
| Unchanged – same services before and during | N/A | 57 | N/A | 56 | N/A | 43 | N/A | 33 |
