# Supplementary material for: Lessons from the COVID-19 pandemic to strengthen NCD care and policy in humanitarian settings: a mixed methods study exploring humanitarian actors’ experiences
Source: BMC Health Serv Res. 2024 Sep 17;24:1081. doi: 10.1186/s12913-024-11458-2 (PMC11406764; doi:10.1186/s12913-024-11458-2)
Supplement: Supplementary file 4 — Supplementary Material 4. [file 12913_2024_11458_MOESM4_ESM.docx]

| **Annex 4: Challenges experienced in delivering NCD care during the COVID-19 pandemic and adaptations made** | | |
| --- | --- | --- |
|  | | |
|  |  |  |
| **If you had to stop or suspend any of the DM/HTN services that were provided before the pandemic, why was this necessary?** | **No.** | % (n=98) |
| We did not suspend DM/HTN services due to the pandemic | 74 | 76 |
| Insufficient lay staff to support routine care | 7 | 7 |
| Insufficient medical staff to support routine care | 14 | 14 |
| Interrupted medication supply/ stock outs | 11 | 11 |
| Insufficient personal protective equipment (PPE) | 14 | 14 |
| Disruption to physical access to services | 17 | 17 |
| Movement restricted due to government guidelines | 32 | 33 |
| Poor internet coverage | 8 | 8 |
| Poor phone coverage | 4 | 4 |
| PLWNCDs fear of face-to-face attendance | 23 | 24 |
| Stopped temporarily while adaptations made | 17 | 17 |
| Don't know | 0 | 0 |
| Other | 6 | 6 |
| **Challenges PLWNCDs faced in managing their DM/HTN that were due to or exacerbated by the pandemic** |  |  |
| Physical restriction/ restricted movement | 81 | 83 |
| Social restriction | 59 | 60 |
| Financial hardship | 48 | 49 |
| Limited healthy food options | 41 | 42 |
| Limited access to medical consultation | 35 | 36 |
| Limited access to medicines | 26 | 27 |
| Inability to access disease monitoring | 25 | 26 |
| Poor mental health (e.g., depression) | 41 | 42 |
| Don't know | 1 | 1 |
| Other | 3 | 3 |
| Distrust of information related to pandemic | 33 | 34 |
| Distrust of health services | 17 | 17 |
| Fear of attending healthcare services | 53 | 54 |
| **Internal / programmatic challenges from the provider perspective** |  |  |
| Medication procurement/supply issues | 44 | 45 |
| Staff workload/burnout | 48 | 49 |
| Staff absence due COVID diagnosis or related quarantine/isolation | 59 | 60 |
| Limited PLWNCDs physical access/movement restrictions | 58 | 59 |
| PLWNCDs fear restricting attendance at facility based appointments | 57 | 58 |
| Unequal access to care; certain groups (e.g., women or elderly) are struggling to access care more than others | 25 | 26 |
| Inability to monitor glucose remotely | 39 | 40 |
| Inability to monitor blood pressure remotely | 39 | 40 |
| Uncertainty making planning difficulty | 26 | 27 |
| Violence against staff | 4 | 4 |
| Don't know | 1 | 1 |
| Other | 2 | 2 |
| Health resources diverted to COVID pandemic | 26 | 27 |
| **External/contextual challenges** |  |  |
| Financial implications of crisis | 60 | 61 |
| Poor mobile phone coverage (non-smartphone) | 27 | 28 |
| Poor Smartphone availability among PLWNCDs and health providers | 34 | 35 |
| Internet connectivity issues | 34 | 35 |
| Resistance among PLWNCDs to adapt to telephone or video consultations | 22 | 22 |
| Don't know | 20 | 20 |
| Other | 4 | 4 |
| **Adaptations made to deliver care for DM/HTN, or to facilitate PLWNCDs self-management DURING the pandemic** |  |  |
| Reduced frequency of face-to-face medical consultation | 72 | 74 |
| Adaptations to medications procurement/supply | 31 | 32 |
| Adaptations to medication pick up/ delivery to PLWNCDs | 47 | 48 |
| Task sharing of certain tasks to facility-based staff | 25 | 26 |
| Task sharing of certain tasks to community-based staff | 21 | 21 |
| Telephone consultations | 21 | 21 |
| Proactive phone calls using PLWNCDs register | 18 | 18 |
| Use of SMS text messages | 13 | 13 |
| Use of smartphone apps for communication (e.g. WhatsApp) | 21 | 21 |
| Use of other social media (Facebook, Instagram) | 9 | 9 |
| Use of specific DM/HTN apps for PLWNCDs self-management | 4 | 4 |
| Simplification of treatment algorithms | 8 | 8 |
| Simplification/reduced frequency of lab testing/monitoring | 19 | 19 |
| Telemedicine (remote clinical support for health workers) | 9 | 9 |
| Decision support tools for health workers | 6 | 6 |
| Don't know | 1 | 1 |
| Other | 6 | 6 |
